# The Cost of Self‐Sufficiency: Could New Zealand be Self‐Sufficient in Food Consumption?

**DOI:** 10.1002/snz2.70083

**Published:** 2026-10-10

**Authors:** R. W. McDowell, N. W. Smith, C. Eason, C. Smith

**Affiliations:** ^1^ New Zealand Institute for Bioeconomy Sciences Lincoln New Zealand; ^2^ Soil and Environmental Sciences, Faculty of Agriculture and Life Sciences Lincoln University Lincoln New Zealand; ^3^ New Zealand Institute for Bioeconomy Sciences Palmerston North, Christchurch New Zealand; ^4^ Sustainable Nutrition Initiative Riddet Institute, Massey University Palmerston North New Zealand; ^5^ AuOra Ltd, Wakatū Incorporation Nelson New Zealand; ^6^ Kōtātā Insight Ltd. Wellington New Zealand

**Keywords:** food security, food systems, incentive, land use policy, land use suitability, natural resource economics

## Abstract

Ensuring that New Zealand can sustainably meet its future food needs requires an understanding of the interaction between domestic demand trends, trade and the capacity of national production systems. Although New Zealand is a major exporter of dairy, meat, fruit and other primary products, rising food insecurity, changing dietary preferences and increasing exposure to geopolitical and climate‐related shocks have renewed interest in the resilience of domestic food supply. Using national consumption data (2010–2023) and production and yield data (2000–2024), we assessed temporal trends across OECD–FAO food groups and projected consumption and production to 2045. We then quantified land requirements needed to meet projected demand and compared these with the availability of highly and moderately suitable land, alongside a regional economic assessment of land‐use change. Results showed that New Zealand production exceeded domestic demand for most food groups, with projected deficits concentrated in cereals and legumes, nuts and seeds, and smaller shortfalls in fruits. Much of the projected cereal deficit was associated with animal feed demand rather than direct human consumption. The land and economic costs of addressing domestic shortfalls were small, suggesting that land availability is unlikely to constrain future domestic food production or any moves to greater self‐sufficiency. Instead, maintaining food security may depend more strongly on processing infrastructure, storage and distribution networks, market incentives and equitable access to nutritious foods. Although the direct impact of changes in domestic production on food security is limited compared to demand‐side issues, there are potential changes to production, processing and distribution that would improve resilience to external shocks to the food system.

## 1 Introduction

Ensuring that New Zealand can sustainably meet its future food needs requires an understanding of the interaction between domestic demand trends, trade and the capacity of national production systems. Although New Zealand is a globally significant exporter of dairy, meat, fruit and other primary products, producing food consumed by tens of millions of people (KPMG 2015), domestic food security is not guaranteed. Recent reports suggest rising rates of food insecurity in New Zealand, driven largely by affordability issues. While domestic pressures, including a rise in the proportion of the population with low incomes (Statistics New Zealand 2025a) and a lack of competition in the New Zealand supermarket sector (Commerce Commission 2022), play a role in food security concerns, factors external to New Zealand including supply‐chain disruptions and climatic shocks have also recently had a major impact on food affordability in New Zealand (Soliman and Greenhalgh 2023; Lloyd et al. 2024; Barber and Cameron 2025). These pressures, also felt globally (Stehl et al. 2025), highlight the disparity between food output and the reliability, accessibility and diversity of food available to New Zealand consumers.

Demand for different food groups is shifting because of socio‐economic change, urbanisation and evolving dietary preferences. Consumption of processed and convenience foods is increasing, while the proportion of adults and children (<15 years old) eating the recommended intake of vegetables has decreased from 9.0% to 6.8% and from 8.6% to 5.8%, respectively (Mackay et al. 2019; Ministry of Health 2025). Concurrently, interest in plant‐based proteins is expanding (Weerawarna et al. 2024). However, plant‐based products remain a comparatively small part of the protein market relative to animal‐sourced foods. The composition of domestic production is also important, with substantial cereal production directed to livestock feed rather than for human consumption, while dairy and meat remain the dominant export‐oriented sectors.

On the production side, New Zealand's food and fibre sectors have historically demonstrated strong capacity, especially in export‐driven industries such as dairy, red meat, kiwifruit and wine (Ministry for Primary Industries 2025c). However, not all food groups exhibit the same level of resilience. Horticultural production is particularly sensitive to climate variability and relies heavily on high‐quality soils that are increasingly under pressure from urban expansion (Curran‐Cournane et al. 2023; Nixon 2024). Climate change will continue to amplify risks across sectors through more frequent droughts, heavy rainfall events and shifts in pest and disease dynamics (Hopkins et al. 2015; Macinnis‐Ng et al. 2024).

A critical component of assessing the capacity to grow food is the concept of land use suitability (LUS) (McDowell et al. 2018). LUS evaluates whether land is appropriate for a given crop by integrating productive capacity, environmental constraints and socio‐economic feasibility. By applying LUS to major food groups, it is possible to identify areas where domestic production can realistically expand to meet projected demand, as well as regions where constraints may limit growth. This approach provides a more nuanced understanding of production potential than simple total output, ensuring that expansion plans are both productive and sustainable.

As New Zealand seeks to enhance food system resilience, we assess consumption trends over the last two decades, evaluating the capacity of domestic food production systems to meet projected demand and identifying LUS constraints that may limit the availability of key food groups (using the OECD–FAO classification (OECD/FAO 2023)). Our analysis suggests that New Zealand faces increasing domestic production deficits in several food groups, although the quantities involved are small relative to total national production. The level of farm and enterprise revenue foregone by addressing domestic production deficits is relatively small compared to both the size of the food sector and New Zealand government expenditure, but ensuring that the infrastructure exists to support the processing, storage and distribution of diverse crops may represent a larger challenge.

## 2 Materials and Methods

National‐level data on food commodity consumption (tonnes) were obtained from FAO Food Balance Sheet statistics (2010–2023; using the Food element) (Food and Agriculture Organization of the United Nations 2025). Production data (tonnes and hectares in production) were sourced from Statistics New Zealand (2000–2024) (Statistics New Zealand 2025b). Both consumption and production data were classified according to OECD–FAO food groups, which are widely used for comparing agricultural commodity prospects across countries (OECD/FAO 2023) and for assessing self‐sufficiency relative to ideal diets (Stehl et al. 2025). Consumption data did not distinguish between domestically produced and imported commodities.

Food commodities produced were aligned, as closely as possible, with those consumed (Table 1). Some consumed commodities had no production analogue, mainly because they are not grown commercially in New Zealand; these accounted for 16% of food mass consumed in 2025. To account for temporal changes in consumption, production and yield (production per hectare), we first tested the normality of each commodity's data. After confirming that the data were normally distributed (within a commodity), year was treated as a continuous explanatory variable and commodity consumption, production or yield as the response variable; linear regression was then applied to determine whether consumption, production or yield values changed significantly over time. For 2025, we used the regression‐estimated value where a significant (*p* < 0.05) trend was detected or the mean value where no significant trend was present. The year 2025 was selected as the common baseline because it immediately followed the latest complete annual series available for consumption (2023) and production (2024), allowing all commodities to be expressed on a consistent baseline before projection to 2045. Where Statistics New Zealand did not provide production or yield data, we used values from published or grey literature (all post‐2009; Table 1).

**TABLE 1:** Organisation for Economic Co‐operation and Development and the United Nations Food and Agriculture Organization food groups (OECD/FAO 2023) classified further by FAOSTAT (Food and Agriculture Organization 2020) consumption and Statistics New Zealand production analogue (Statistics New Zealand 2025b) for New Zealand. References are given for production statistics and any conversion necessary to calculate annual production in tonnes if not sourced from FAOSTAT or Statistics New Zealand.

OECD–FAO food group	FAOSTAT annual consumption, tonnes	Statistics New Zealand production analogue, tonnes	Production reference, if not Statistics New Zealand^a^
Cereals	Barley	Barley	—
	Maize	Maize (corn)	—
	Oats	Oats	—
	Rice	—b	—
	Wheat	Wheat	—
	Other cereals	Other cereals	—
Meat	Bovine	Cattle meat—chilled	—
	Eggs	Hen eggs	—
	Mutton and Goat	Sheep meat—chilled	—
	Pig	Pig—chilled	—
	Poultry	Chicken—chilled	—
	Other meat	Duck, game meat, goat, geese, turkey	—
Fish and seafood	Cephalopods	Squid	Ministry for Primary Industries (2025b),
	Crustaceans	Rock lobster and giant spider crabs	Ministry for Primary Industries (2024b), New Zealand Rock Lobster Industry Council (2025)
	Demersal fish	Demersal fish	FAO (2020)
	Freshwater fish	—	
	Molluscs	Greenshell mussels and Pacific oysters	Stenton‐Dozey et al. (2021)
	Pelagic fish	Pelagic fish	FAO (2020), Ministry for the Environment (2021)
	Other marine fish	—	
Dairy	Butter and ghee	Calculated from milk production	Dairy Food Safety Australia (2025)
	Cream	Calculated from milk production	Produits Laitiers de France (2025)
	Milk	Raw milk of cattle	—
Legumes, nuts and seeds	Coconuts—including copra, coffee, cottonseed oil, groundnuts, nuts, sesame seed, sesame seed oil, soybean oil, soybeans	—	—
	Maize germ oil	Calculated from maize (corn)	Stock et al. (2000)
	Olive oil	Olive oil	Sandua (2021), AZUD (2023)
	Olives	Calculated from olive oil production	—
	Peas	Peas	—
	Rape and mustard oil	Calculated from rape or colza seed	Manitoba Canola Growers (2023)
	Other oil crops	Linseed	—
	Other pulses	Lentils	—
Fruits	Apples	Apples	—
	Bananas, pineapples	—	—
	Grapefruits	Pomelos and grapefruits	—
	Grapes	Grapes	—
	Honey	Natural honey (manuka, clover)	—
	Oranges, mandarins	Clementines, tangerines, mandarins, oranges	—
	Tomatoes	Tomatoes	—
	Other citrus	Other citrus	—
	Other fruits	Apricots, avocados, blueberries, cantaloupes, melons, currants, gooseberries, kiwifruit, pears, persimmons, plums, sloes, quinces, raspberries, strawberries, watermelons, other stone fruits, other tropical fruits.	—
Vegetables	Plantains	—	—
	Potatoes	Potatoes	—
	Sweet potatoes	Sweet potatoes	—
	Yams	Sweet potatoes	—
	Other vegetables	Asparagus, cabbages, chillies, cucumber and gherkins, cauliflowers, green garlic, lettuce and chicory, mushrooms, onions and shallots, spinach, other fresh vegetables	—

a Conversions from raw analogue into class produced. Butter: assumes 20 L of milk is required to make 1 kg of butter (Dairy Food Safety Australia 2025). Cream: assumes 7 L of raw milk is required to make 1 L of cream product containing 30% fat (Produits Laitiers de France 2025). Maize germ oil: assumes 11% of the maize kernel is maize germ and it contains about 50% oil (Stock et al. 2000). Olive oil: assume 7.5 kg of Olives are required to make 1 L of oil (Sandua 2021). Rape and Mustard Oil: assume 1 kg of Rape seed contains 43% oil (Manitoba Canola Growers 2023).

b No analogue exists and class not considered.

For comparability between consumption and production, production values were adjusted for estimated losses during harvesting, post‐harvest handling and storage, processing and packaging and retail distribution (Gustavsson et al. 2011) (Table 2). The FAO Food Balance Sheet statistics already represent food available for human consumption.

**TABLE 2:** Percentage food losses (Gustavsson et al. 2011) associated with each stage of the value chain from production.

OECD–FAO food group	Agricultural production	Post‐harvest handling and storage	Processing and packaging	Distribution: Supermarket Retail	Total losses excluding production
Cereals	2	2	5	2	14%
Meat	4	1	5	4	10%
Fish and seafood	12	1	6	9	16%
Milk	4	1	1	1	2%
Legumes, nuts and seeds	12	0	5	1	6%
Fruits	20	4	2	12	18%
Vegetables	20	4	2	12	18%

### 2.1 Scenario Analysis

#### 2.1.1 Production and Consumption Projections

National production for each commodity was calculated for 2025 and 2045 using either mean values or regression‐estimated trends from historical data. Production estimates were adjusted for post‐harvest food losses and aggregated into OECD–FAO food groups. Because these groups contained multiple commodities with varying growth rates and production volumes, group‐level projections were obtained from the summed constituent‐commodity projections, so larger‐volume commodities contributed proportionally more to the food‐group result rather than each commodity receiving equal weight. Consumption was calculated using the same temporal approach but without loss adjustments. The 20‐year projection interval was selected to represent a demographic generation shift while maintaining a reasonable forecasting horizon, given the constraints of available historical data. Population growth used in the sensitivity analysis was taken from Statistics New Zealand's National population projections: 2024 (base) − 2078 (published 4 June 2025), using the median projection. The population was 5.34 million in 2025 and 6.38 million in 2045; the 2045 value was obtained by linear interpolation between the published 2043 and 2048 median projections. For the constant‐per‐capita sensitivity scenario, 2025 food consumption was scaled by this population change while holding consumption per person constant.

We benchmarked the production of each food group against that recorded by Statistics New Zealand for 2023 (Statistics New Zealand 2025b), highlighting groups where projected consumption exceeded production, indicating that food would likely have to be imported to meet consumption.

#### 2.1.2 Land Use Requirements and Suitability Assessments

Land requirements for the 2025 and 2045 scenarios were calculated using mean or trend‐based yields where significant temporal trends (*p* < 0.05) were detected. These calculations assume commodities are sourced from livestock or perennial crops or from annual crops (such as cereals) grown at least once every 2 years within cropping rotations. However, some vegetable crops may be cultivated on longer rotations, potentially resulting in lower actual land requirements than those calculated. Our land use estimates should therefore be interpreted as conservative upper bounds for vegetable production.

Projected land use for each food group was compared against both Statistics New Zealand 2023 data (Statistics New Zealand 2025b) and the available area of highly suitable land for production (see Figures S1–S16). Highly suitable land was defined as having either a suitability score ≥ 0.8 (on a scale where 1.0 represents optimal conditions) or a ranking of 1 on a five‐tier scale (representing the top 20th percentile of suitability) (McDowell et al. 2018; Harris et al. 2021). Because suitability scores were available for only a subset of consumed commodities, we assigned the most closely aligned crop with available suitability data as a proxy for other foods within each group (Table 3). Where consumption‐driven land requirements exceeded the available highly suitable area, the resulting deficit indicated production occurring on suboptimal (moderately suitable) land. We used quotas and typical catch rates to calculate production per hectare for fish and seafood and did not consider onshore aquaculture. We assessed these deficits at both national and regional scales to provide geographic context.

**TABLE 3:** Alignment of crops with suitability scores to food groups of the Organisation for Economic Co‐operation and Development and the United Nations Food and Agriculture Organization (OECD/FAO 2023).

OECD–FAO food group	Proxy crops with suitability scores included in group	Suitability references	Gross margin, $ NZD ha^−1^ yr^−1^ (McDowell et al. 2022)^a^
Cereals	Barley, maize, oats, wheat	Thomas et al. (2021)	2309 (mean of barley, oats and wheat)
Meat	Sheep and beef, pork and dairy	Harris (2024)	—
Fish and seafood	—b	Ministry for Primary Industries (2024a)	—
Milk	Dairy	Harris (2024)	3759 (Waikato), 5262 (Canterbury), 3954 (Southland)
Legumes, nuts and seeds	Peas, chestnuts, macadamias, walnuts	Thomas et al. (2021), Thomas (2023a)	1955 (mean of beans and peas)
Vegetables	Onions, peas, potatoes	VetharaniamI et al. (2022), Thomas (2023b)	6501 (mean of carrots, onions and potatoes)
Fruits	Apples, avocados, blueberries, cherries, citrus, kiwifruit	VetharaniamI et al. (2022), Thomas (2023b)	15,822 (mean of apples and citrus)

a Gross margins were sourced from Reynish (Reynish 2020) for dairy, wheat, barley, potatoes, and onions; the margin for apples were sourced from the Pipfruit Monitoring Programme (Ministry for Primary Industries 2022); the margin for oats came from the Foundation for Arable Research, while Horticulture New Zealand supplied margins for peas, carrot, citrus and beans. Potato data are for the Agria variety.

b Suitability for fish and seafood assumed as equal to the catch permissible under the New Zealand Quota Management System (Ministry for Primary Industries 2024a) with areas calculated from the yield per hectare caught as outlined in the references given in Table 1.

#### 2.1.3 Regional Economic Analysis

We evaluated potential changes in regional agricultural profitability associated with meeting national production deficits by assuming that shortfalls in cereals, legumes, nuts and seeds, and fruits (excluding kiwifruit) would be met through land‐use conversion from dairying, a land use widespread in each region whose land would likely be suitable for many other uses. For both 2025 and 2045, the required converted area was distributed across regions in proportion to each region's available highly suitable land for the relevant food groups. Profitability indicators were derived from published gross margin estimates and averaged across food groups (Table 3) (McDowell et al. 2022). These data incorporate some regional climate and biophysical variation for dairy systems, including bespoke values for Canterbury (under irrigation), with Southland data applied to Southland, Otago and the West Coast and Waikato data applied to all remaining regions. However, equivalent regional variation for cereals, legumes, nuts and seeds, and fruit production was unavailable. We also applied constant 2025 profitability values to the 2045 projections. Although this does not capture potential temporal changes in profitability, it allows for a consistent comparison of relative differences across land uses at both time points.

## 3 Results

### 3.1 National Food Production and Consumption Trends

Analysis of New Zealand's food system reveals that current production exceeds consumption for most food groups, with the notable exceptions of cereals and legumes, nuts and seeds (Figure 1). Projecting these trends to 2045 shows a shift in New Zealand's food self‐sufficiency profile. In 2025, only one food class (legumes, nuts and seeds) requires import support. However, projections for 2045 indicate a deterioration in self‐sufficiency, with fruits (except kiwifruit) and cereals also becoming dependent on imports (Figure 2). This trend is particularly significant for cereals given that 50%–80% of cereal production is allocated to animal feed depending on harvest quality (a dynamic not captured in our analysis) (Ministry for Primary Industries 2025a).

**FIGURE 1:**
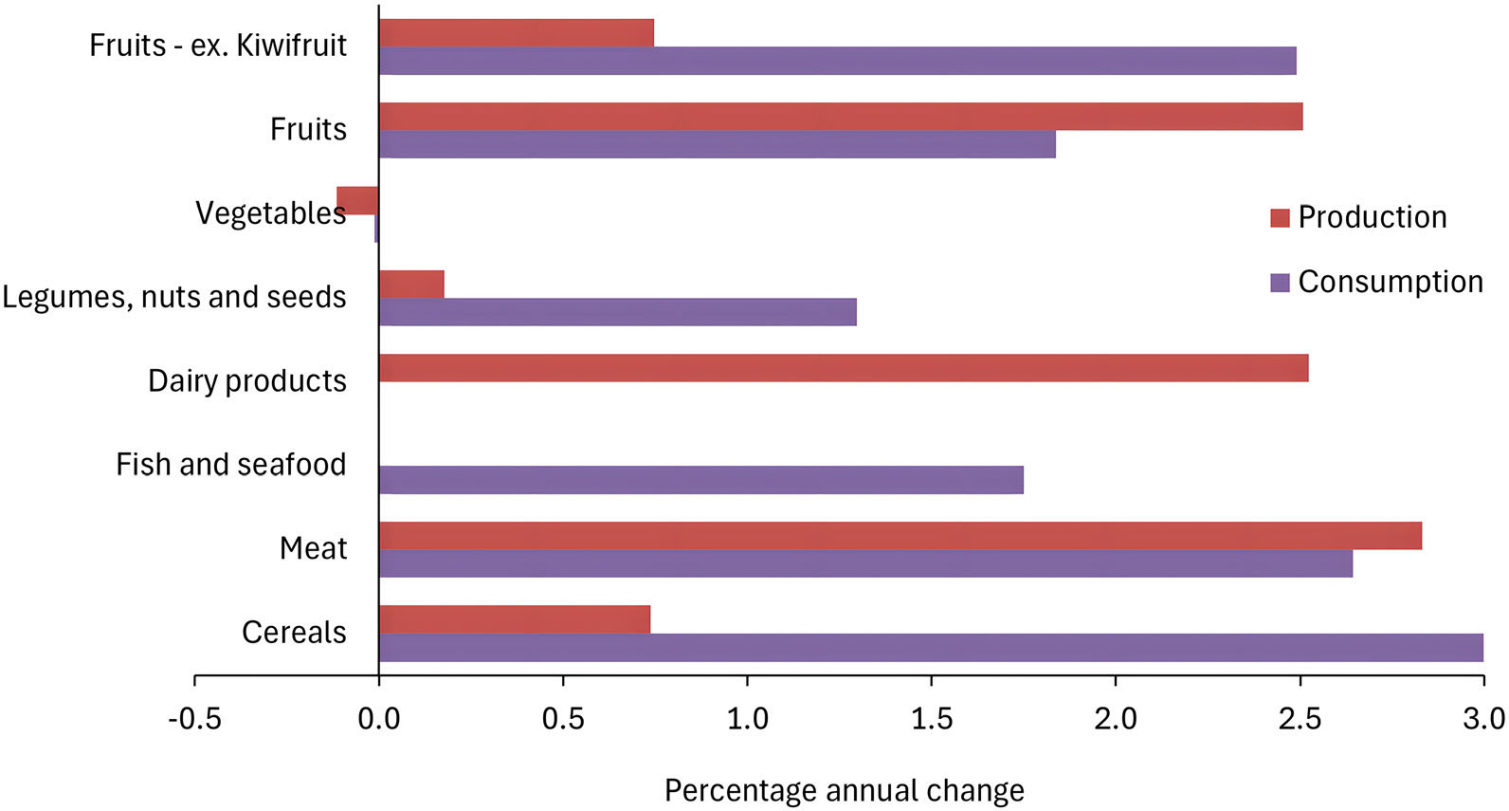
Magnitude of significant ( *p* < 0.05) linear trends in the consumption since 2010 or production since 2000 of OECD–FAO food groups in New Zealand. Trends for consumption greater than production indicates a decline in net production. Food groups do not contain those not commercially grown in New Zealand and are fully imported (bananas, cassava, cocoa beans, coconuts, coffee, cottonseed oil, groundnuts, pepper, pimento, pineapples, plantains, rice, sesame seed or oil, soybean or oil, sunflower oil, various spices, sugar, other sweeteners and tea).

**FIGURE 2:**
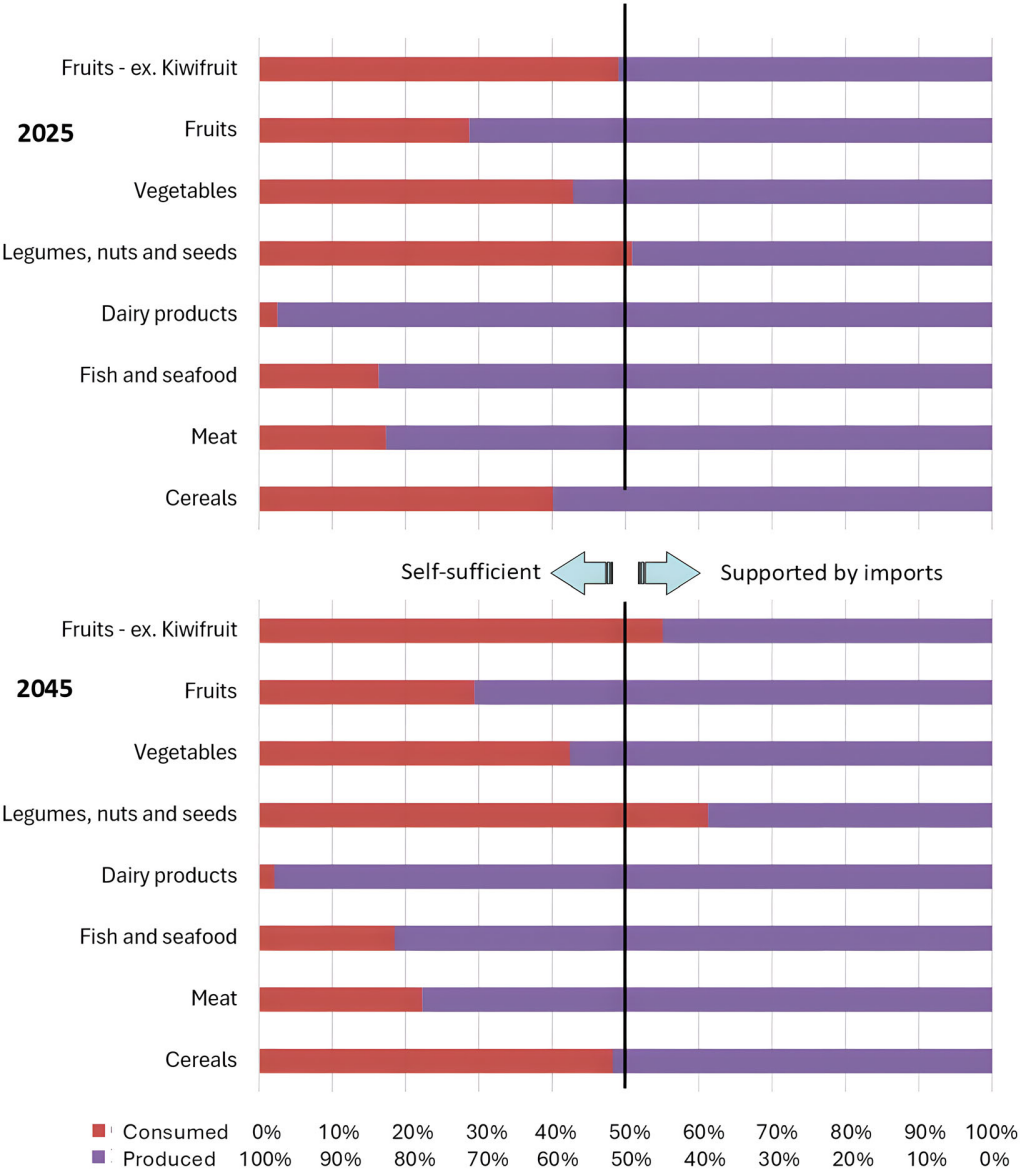
Relative cumulative proportion of OECD–FAO food groups produced and consumed in New Zealand in 2025 and estimated for 2045 using trends for production since 2000 and consumption since 2010. A proportion less than 50% indicates self‐sufficiency with more being produced than consumed. Food groups do not contain those not commercially grown in New Zealand and are fully imported (bananas, cassava, cocoa beans, coconuts, coffee, cottonseed oil, groundnuts, pepper, pimento, pineapples, plantains, rice, sesame seed or oil, soybean or oil, sunflower oil, various spices, sugar, other sweeteners and tea).

### 3.2 LUS and National Deficits

The divergence between consumption and production trends results in an increasing deficit of highly suitable agricultural land required to produce all food classes. At the national level, deficits for 2025 and 2045 can be accommodated by highly suitable land for all food groups except cereals, oilseeds and pulses (Table 4). This necessitates one or more alternative approaches: imports, the utilisation of less‐suitable land, land use change or modification of current land use practices (e.g., using less cereal production for animal feed).

**TABLE 4:** National area of land by food groups of the Organisation for Economic Co‐operation and Development and the United Nations Food and Agriculture Organization that is in deficit for 2025 and 2045 relative to the area of highly‐suitable land.

OECD–FAO food group	2025 Deficit, ha	2045 Deficit, ha	Area of highly‐suitable land, ha
Cereals	14,151	23,654	5679
Meat (pork)	4254^a^	7225^a^	~229,900^a^
Fish and seafood	817^b^	1418	13,044
Milk	—c	—	~1,700,000^d^
Legumes, nuts and seeds	25,662	43,082	16,509
Vegetables	95	1008	1250
Fruits	4614	6118	107,423

a Perceived deficit only for pig meat. Highly suitable land taken as equivalent to the current area in class 8 farms by Beef and Lamb New Zealand (South Island Mixed finishing farms Sinnamon et al. 2025) in 2025, as having moderate climate, low rainfall and free‐draining soil suitable for outdoor pigs (Smart Shelters 2025).

b The deficit recorded for fish and seafood reflects an imbalance in production quota and demand.

c No deficit recorded.

d Equivalent to the current area used for dairying (Livestock Improvement Corporation and DairyNZ 2024).

For 2025, the most substantial land deficits occur in legumes, nuts and seeds (25,662 ha), followed by cereals (14,151 ha), despite the existence of approximately 16,509 ha of highly suitable land for production of legumes and 5679 ha available for cereals. By 2045, these deficits are projected to increase to 43,082 ha for legumes, nuts and seeds and 23,654 ha for cereals. Vegetables and fruits show comparatively modest deficits (95 ha and 4614 ha in 2025, increasing to 1008 ha and 6118 ha in 2045, respectively), despite there being substantial, highly suitable land available for these crops (1250 ha for vegetables and 107,423 ha for fruits). Notably, there is a large surplus in milk production, with approximately 1,700,000 ha of highly suitable land (equivalent to current dairying area). Deficits were noted for meat, but this was caused by a lack of pig meat production, which could easily be met by land use change from sheep and beef production; for example, there is approximately 229,900 ha of highly suitable flat land available in Tasman, Canterbury and Otago alone (Table 5).

**TABLE 5:** National area of land (ha) by food groups of the Organisation for Economic Co‐operation and Development and the United Nations Food and Agriculture Organization that is in deficit for 2025 relative to the area of highly‐suitable land, and moderately‐suitable land in parentheses, in each regional authority: note Tasman is combined with Marlborough and Nelson regions.

OECD–FAO food group	Cereals	Meat (pork)^a^	Fish and seafood^b^	Milk^c^	Legumes, nuts and seeds	Vegetables	Fruits
2025 deficit, ha	14,151	4254	817	—d	25,662	95	4614
Auckland	252 (1565)	—	3180	46,786	266 (4159)	79 (2772)	2316 (5804)
Bay of Plenty	1077 (3074)	—	222	74,296	1541 (3915)	81 (2270)	10,751 (7902)
Canterbury	13 (3574)	68,970	687	349,671	150 (10,848)	7 (9954)	12,874 (23,072)
Gisborne	44 (1896)	—	215	1790	1030 (2376)	12 (871)	4847 (10,514)
Hawke's Bay	151 (2528)	—	301	19,754	587 (5678)	152 (4000)	8865 (12,340)
Manawatu‐Wanganui	171 (3855)	—	137	120,393	1113 (8760)	235 (8594)	13,737 (23,782)
Northland	24 (2894)	—	2749	129,908	544 (7087)	21 (5506)	2755 (13,623)
Otago	6 (876)	55,594	412	120,016	278 (2867)	34 (2266)	925 (8978)
Southland	124 (2750)	—	2921	270,256	2101 (6438)	1164 (7390)	306 (8677)
Taranaki	0 (3822)	—	226	203,485	2037 (2344)	0 (3792)	8186 (6441)
Tasman	41 (940)	105,336	2221	33,606	150 (1960)	102 (1254)	5034 (9822)
Waikato	3968 (9052)	—	1031	459,906	6500 (14,262)	2167 (13,842)	27,251 (21,893)
Wellington	94 (1154)	—	447	68,257	196 (2319)	148 (2792)	5686 (7270)
West Coast	14 (581)	—	515	76,214	16 (2125)	0 (15)	3890 (8022)
Total	5979 (38,561)	229,900	15,265	1,974,336	16,509 (75,138)	4202 (67,980)	107,423 (168,140)

a Highly and moderately suitable land taken as equivalent to the current area in class 8 farms by Beef and Lamb New Zealand (South Island Mixed finishing farms Sinnamon et al. 2025) in 2025, as having moderate climate, low rainfall and free‐draining soil suitable for outdoor pigs (Smart Shelters 2025).

b Taken as the regional proportion of the national suitable area (via quota) calculated using each regions coastline.

c No deficit assumed and area assumed equivalent to the regional proportion of land used by dairy as of 2023/24 season (Gerrard 2024).

d Not applicable.

### 3.3 Sensitivity of Results to Consumption Modelling

To better understand the projected land deficits, we decompose projected consumption over the 2025–2045 forecast period into changes due to population growth (i.e., the number of people in New Zealand) and changes in consumption per capita (i.e., how much of each food group is consumed per person). This analysis is presented in Figure 3 and highlights that the modelling assumes relatively strong growth in per capita consumption of cereals and meat over the 2025–2045 period, accompanied by decreases in per capita consumption of dairy and vegetables. These changes in per capita consumption are non‐trivial, implying cereal consumption per head increases by 22% over the period and meat consumption per head by 19%. Conversely, dairy consumption per head falls by 16% and vegetable consumption per head by 17%.

**FIGURE 3:**
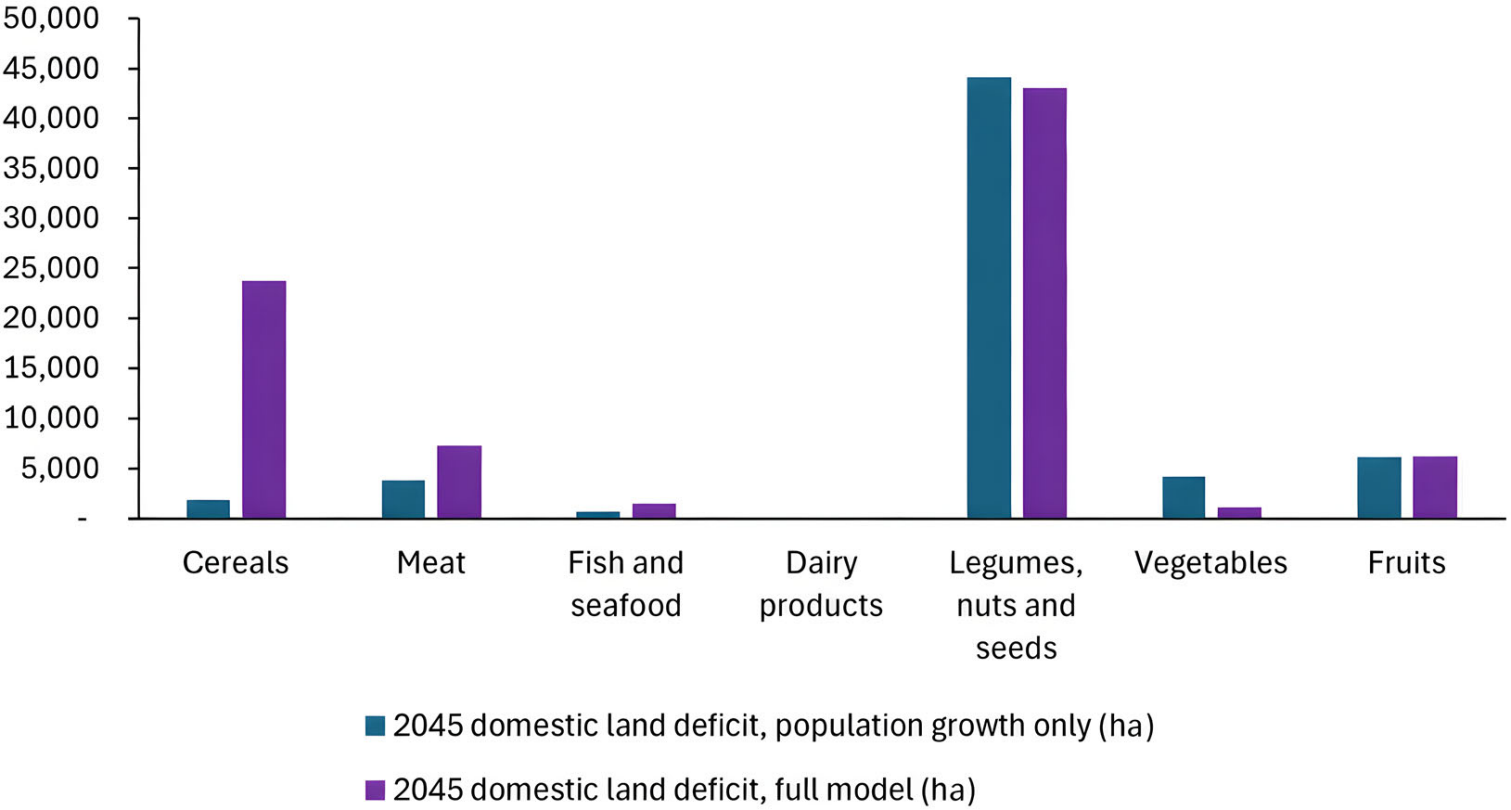
Changes in projected food consumption (tonnes), 2025 to 2045, decomposed into changes due to population growth and changes due to consumption per head.

To test the sensitivity of our results, we compare food production and consumption in 2045 while holding constant consumption per capita (Table 6). This reduces the land area deficit for cereals and meat but increases it for vegetables. In particular, the land deficit for cereals in 2045 is only 1758 ha if per capita consumption remains constant at 2025 levels compared to the 23,654 ha reported for 2045 if consumption is assumed to increase in line with trends over the decade to 2024. In contrast, the 2045 land deficit for vegetables is 4134 ha if consumption per capita remains constant but only 1008 ha if current trends in consumption per capita continue until 2045. The total national land deficit for 2045 across all seven major food categories reported in Table 6 is 60,571 ha if consumption per capita is assumed to remain constant compared to 82,505 ha in the full model where consumption per head is allowed to change. These 2045 estimates should therefore be interpreted as a continuation of these trends rather than forecasts of individual dietary intake. They assume that recent aggregate and per‐capita trajectories persist, whereas prices, dietary preferences, demographic composition, policy and substitution among food groups could alter those trajectories.

**TABLE 6:** Projected national area of land (ha) by food groups of the Organisation for Economic Co‐operation and Development and the United Nations Food and Agriculture Organization that is in deficit for 2045 based on constant consumption per capita and modelled changes to consumption per capita (full model).

Food group	2045 Domestic land deficit, constant consumption per capita, ha	2045 Domestic land deficit, full model, ha
Cereals	1758	23,654
Meat	3821	7225
Fish and seafood	681	1418
Dairy products	—	—
Legumes, nuts and seeds	44,066	43,082
Vegetables	4134	1008
Fruits	6111	6118
Total	60,571	82,505

### 3.4 Regional Distribution of Production Capacity

Regional analysis reveals considerable heterogeneity in the capacity to address national food production deficits (Table 5). The Waikato region demonstrates the greatest flexibility to accommodate deficits across multiple food groups, with particularly large areas of highly suitable land. Canterbury, Manawatu‐Wanganui and the combined Tasman‐Marlborough‐Nelson region also possess abundant highly suitable land, particularly for fruits and legumes and pulses production.

Specific regional patterns emerge when examining individual food groups. For cereals, most regions show modest deficits when considering only highly suitable land, with substantially larger areas of moderately suitable land (in parentheses in Table 5). The Waikato region exhibits the largest cereal deficit at 3968 ha of highly suitable land. For meat production, surplus highly suitable land exists in several regions, with Canterbury (68,970 ha), Northland (105,336 ha), Otago (55,594 ha) and Tasman (105,336 ha) showing particularly large areas available. Fish and seafood production capacity, distributed according to regional coastline proportions, shows Auckland (3180 ha) and Southland (2921 ha) with the largest allocations.

The distinct geographical patterns in our ability to mitigate deficits are also shown in Figures 4 and 5. For cereals and legumes, nuts and seeds, certain regions possess proportions of suitable land exceeding the national deficit (regional proportion >1.0), indicating their potential to compensate for shortfalls elsewhere. Similarly, for vegetables and fruits, the distribution of highly and moderately suitable land varies considerably across regions, with some areas showing substantial capacity to address national deficits while others have limited or no suitable land.

**FIGURE 4:**
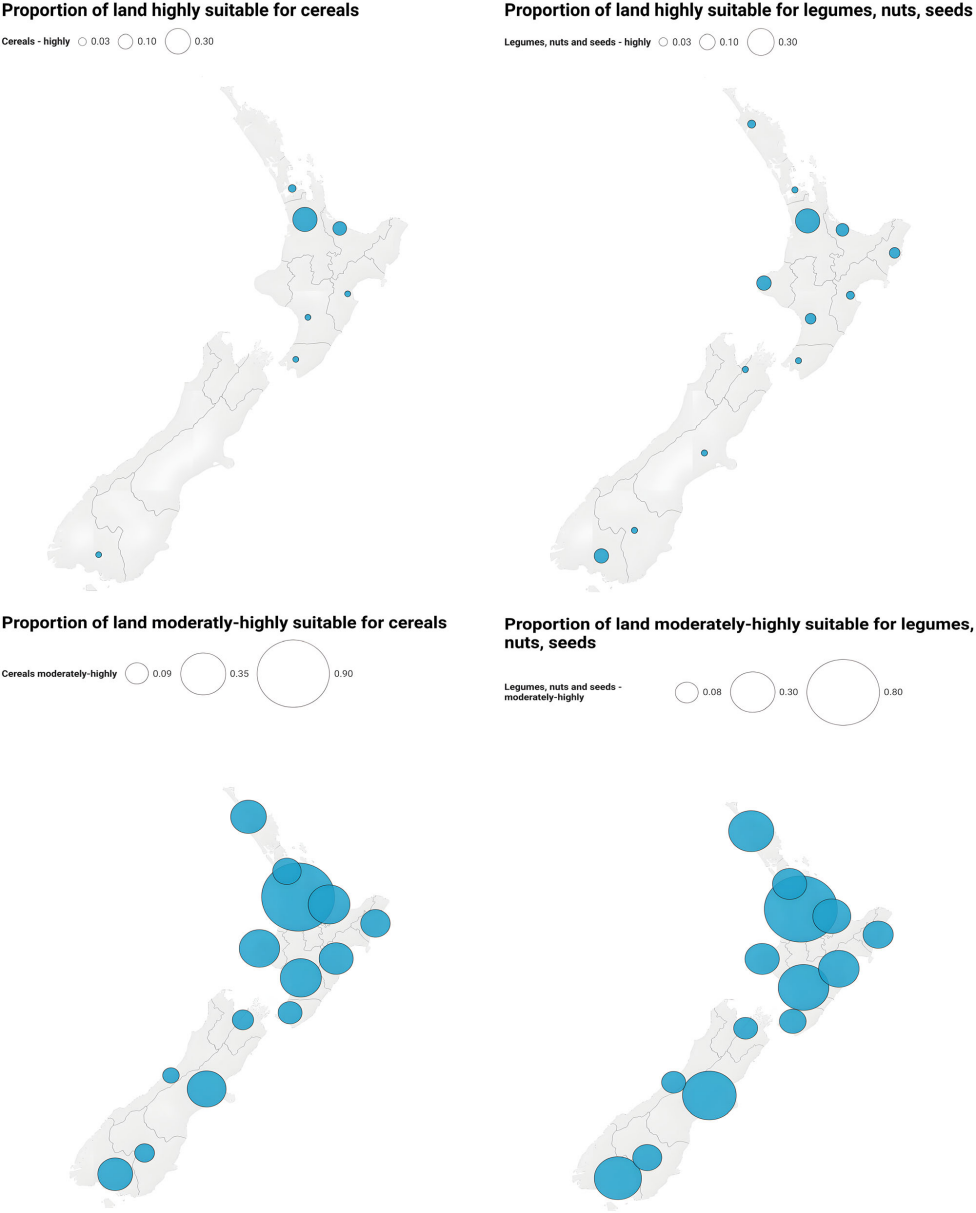
Proportion of highly or moderately and highly suitable land by region available to alleviate the national deficit of cereals and legumes, nuts and seeds. A regional proportion of 1.0 equates to a land area equivalent to the national deficit. No symbol means, no land was suitable.

**FIGURE 5:**
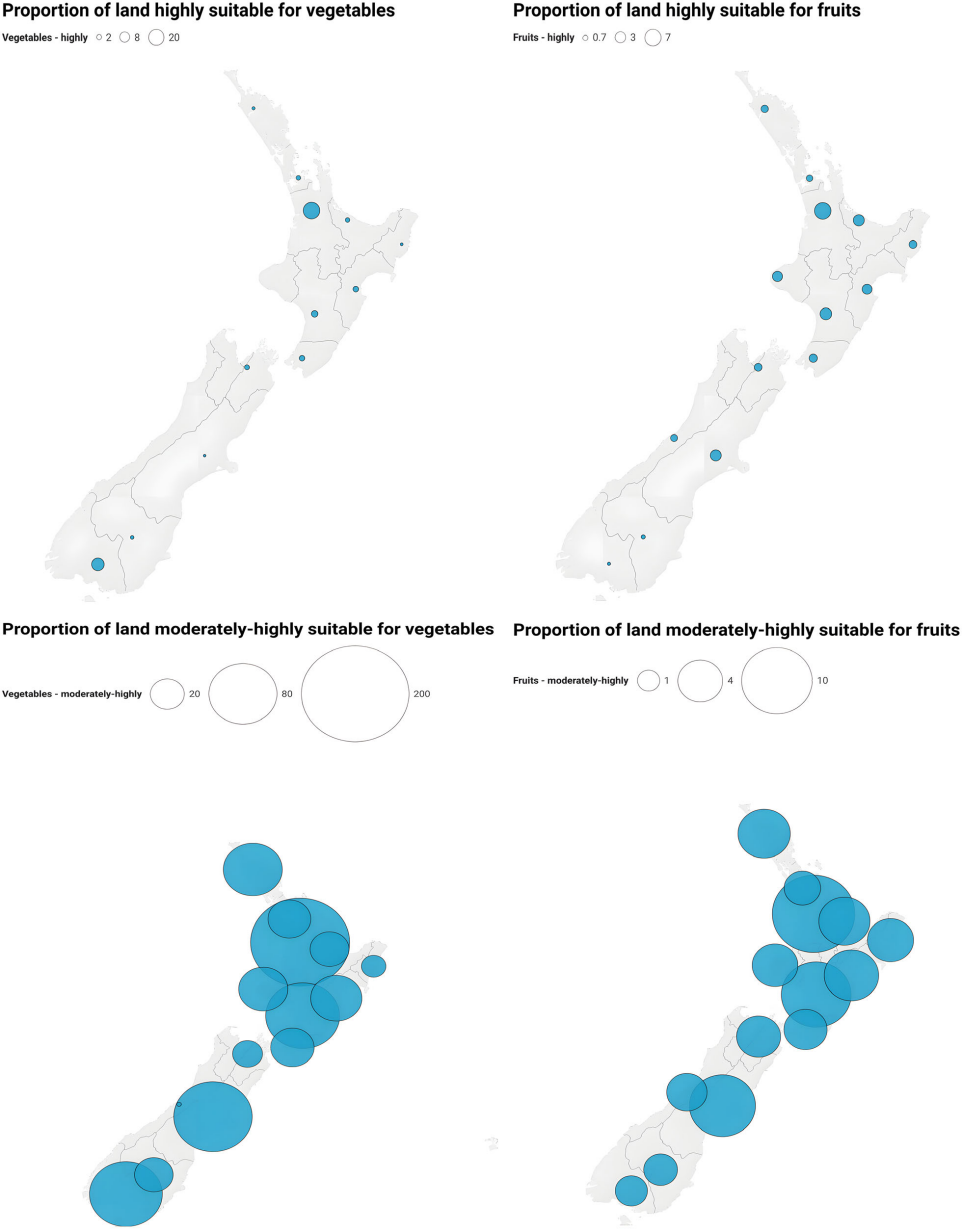
Proportion of highly or moderately and highly suitable land by region available to alleviate the national deficit of vegetables and fruits. A regional proportion of 1.0 equates to a land area equivalent to the national deficit. No symbol means, no land was suitable.

### 3.5 Economic Impact

To explore the economic ramifications of shifting land use, we compared the economic value of current land uses with that needed to meet forecast national consumption of cereals, legumes, nuts and seeds, and fruits to meet demand, at the expense of dairying. Gross margin comparisons (Table 7) show that, in most regions, replacement land uses generate lower profitability per hectare than that of dairying, resulting in lower receipts to farmers under both 2025 and 2045 scenarios. However, the magnitude of these losses to farmers is modest, amounting to less than 0.1% of the contribution of agriculture to the New Zealand economy (Statistics New Zealand 2025b), less than 0.02% of food and fibre sector export revenue for 2025 (Ministry for Primary Industries 2025d) and approximately 3.7% of 2024 estimated New Zealand government producer support to the agricultural sector (OECD 2025). Canterbury showed a particularly marked increase in the estimated net gross‐margin cost, from NZ$0.83 million in 2025 to NZ$8.96 million in 2045 (Table 7), reflecting the larger land reallocation required in the 2045 scenario and the relatively high dairy gross margin used for irrigated Canterbury.

**TABLE 7:** Regional and national net change in gross margin ($ NZD) associated with a change in land use from dairying to meet the deficits for cereal, legumes nuts and seeds, and fruits in 2025 and 2045. The values in parentheses are the losses associated with the change out of dairying without the gains realised from cereal, legumes nuts and seeds, and fruits.

Region	Change in gross margin 2025	Change in gross margin 2045
Auckland	−860,809 (5,472,237)	−2,427,016 (10,668,409)
Bay of Plenty	−407,365 (15,818,387)	−1,006,179 (22,643,733)
Canterbury	−834,097 (14,706,421)	−8,958,445 (33,523,468)
Gisborne	−412,863 (7,418,120)	−1,070,500 (11,500,263)
Hawke's Bay	1,535,541 (8,819,294)	220,642 (16,576,015)
Manawatū‐Whanganui	2,129,792 (14,126,703)	127,470 (26,055,047)
Northland	−1,729,833 (8,130,525)	−4,681,101 (17,132,661)
Otago	−973,969 (3,394,991)	−2,268,804 (6,927,962)
Southland	−6,496,678 (14,254,900)	−10,015,974 (22,853,200)
Taranaki	−1,021,855 (13,096,778)	−1,793,043 (19,110,951)
Tasman	1,643,145 (3,177,056)	1,498,553 (6,020,891)
Waikato	−8,660,867 (57,490,710)	−12,087,487 (79,739,548)
Wellington	1,691,958 (3,989,331)	1,460,328 (7,378,588)
West Coast	1,299,548 (2,289,637)	899,953 (5,019,103)
Nationally	−13,098,351 (172,185,090)	−40,101,605 (285,149,839)

## 4 Discussion

### 4.1 Can We Grow What Our Population Demands?

New Zealand produces and exports about $53.3B NZD (in 2025) of dairy, meat, seafood and fruits that would meet population demand for these foods many times over (Ministry for Primary Industries 2025d). This surplus capacity suggests that, at least in principle, New Zealand easily has the production base to support domestic food security and buffer against global supply‐chain disruptions.

However, the picture becomes more complicated when we consider the structure of agricultural production and current demands. Cereal grains, especially wheat, remain a weak point in domestic production (currently returning $340M NZD) (Ministry for Primary Industries 2025d). According to recent assessments, the proportion of domestically grown cereals used for human food (milling wheat, flour and bread) is small compared to that used for animal feed, with the balance of milling wheat and flour imported largely from Australia (Millner and Roskruge 2013). For example, in 2021, 348,014 tonnes of wheat were used for animal feed and 114,836 tonnes were used for human consumption (Robertson and Hurren 2022). This imbalance is driven by price signals: feed wheat prices track dairy payouts, which have historically been high, while milling wheat prices reflect more volatile global markets. Additional constraints include limited milling capacity (~100,000 t/y), insufficient grain cleaning and silo infrastructure and the high cost of transporting grain from Canterbury (where most wheat is grown) to North Island baking centres. Targeted development of high‐value heritage grains may help overcome some of these constraints as these smaller‐volume crops can be better matched to suitable soils and climatic niches (McLeod et al. 2023), but these will always be competing for market share with imported commodity grains.

Our results indicate that greater diversity in land uses and food groups can be easily achieved by prioritising highly suitable land for vegetable production. However, projected deficits for cereals, legumes, nuts and seeds in 2045 require more than twice the area of highly suitable land estimated in 2025 (Table 4), necessitating production on moderately suitable land or substantial reallocation of land between uses (e.g., from dairy). Some efficiency gains could partially offset these pressures, particularly through reducing food waste, estimated at approximately 5%–10% of production in 2024 (Skeaff et al. 2025).

Land use change also entails opportunity costs. The costs presented in Table 6 are minimums based on the reallocation of land away from dairy. While increased cereal production could be accommodated through reductions in dairy area, alternative approaches to increasing land for cereals, legumes, nuts and seeds would involve the use of moderately suitable land and would likely reduce profitability and therefore impose larger economic costs than those shown in Table 6. Moreover, the characteristics that make land only moderately suitable, such as steeper slopes or higher rainfall, may also reduce nutrient‐use efficiency and increase the risk of nutrient losses to water or greenhouse gas emissions compared with highly suitable land (McDowell et al. 2022).

### 4.2 Should We Be Growing What We Consume?

Several commentators on New Zealand's food system highlight that the mismatch between production and dietary demand potentially leaves the country vulnerable to short‐term disruptions in imported wheat, legumes, nuts and seeds (Public Health Advisory Committee 2024). However, because most imports are sourced from Australia, long‐lasting supply interruptions remain unlikely. Even during the COVID‐19 pandemic, disruptions lasted for only weeks. In addition to this, our modelling suggests that total production deficits are relatively small compared to domestic consumption. A large projected production deficit relates to cereals (Table 4); however, much of this increase reflects demand for animal feed rather than direct human consumption, reducing its implications for food security.

However, focusing on total quantity of food produced directs attention away from the key transfer mechanisms between food production, food consumption and food security. It has been long understood that food insecurity is best understood as a function of the inability of people to access food, not of there not being enough food in the first place (Sen 1982). In New Zealand, the two most important factors driving household food insecurity are low household incomes (Carter et al. 2010) and high food prices relative to both other countries and historical New Zealand prices (Commerce Commission 2025). High food prices are, in turn, partly driven by lack of competition in the domestic food market (Commerce Commission 2025). In this context, it is important to understand that domestic food production, as currently organised, does not insulate New Zealand consumers from international food price shocks. This is because external shocks may directly contribute to higher domestic costs of production, as with the impact of recent geopolitical instability in the Arabian Gulf on diesel and fertiliser prices (Oxford Economics 2026) and because international prices for tradeable commodities such as food strongly impact domestic prices through competitive pressure on the decision to export or sell locally.

Perhaps of equal importance to disruption is the availability and consumption of healthy diets. New Zealand has high rates of diet‐related non‐communicable diseases, with limited adherence to national dietary guidelines, which promote diverse diets including whole grains, legumes, vegetables, fruits, lean proteins and dairy (Ministry of Health 2020). Diet quality research among adolescents further shows entrenched inequities: food‐insecure households consume more refined, low‐nutrient foods and fewer core foods such as vegetables, whole grains and protein‐rich foods (Tomita et al. 2025). While these under‐consumed food groups align with underproduced groups highlighted here, it is unclear how greater domestic production of under‐consumed foods would drive their increased consumption.

Beyond food consumption, an increase in the diversity of land use may lead to agricultural employment, which may help bolster the population of rural communities, keeping schools open and improving the mental well‐being of residents (Yule 2022), but there are many caveats around each of these outcomes. Moreover, here, we assessed the capacity of regions to transition to supply national demand, but another interesting question would be their capacity to meet regional demand. Our poor data availability for population food consumption means that any such analysis would currently be limited to assuming that regional consumption patterns match national patterns, but even this may be instructive. Localisation of food systems is the subject of differing opinion (Smith et al. 2025) and any judgement will be heavily dependent on the degree of localisation and the intended goals of any such action. It is necessary to quantify the sustainability of local food sourcing across the many dimensions of sustainability to provide underpinning evidence for how these impacts align with the values and aspirations of the population.

Thus, the question of whether our diets should be domestically or even more locally sourced has economic, logistical and environmental dimensions, as well as clear cultural relevancy, beyond the few outcomes examined here. What we can conclude is that the biophysical suitability exists for such a transition, with a relatively low impact on total national economic value of production.

### 4.3 Potential Solutions

The modelling reported here suggests that gaps between domestic production and consumption of food are generally small relative to New Zealand's total available land area. Similarly, the direct loss of revenue at the farm and enterprise level associated with reallocation of land to meet domestic needs, if this were a policy goal, is small relative to the total size of the agricultural sector, total government expenditure and the New Zealand economy.

If we choose to move domestic production to better align with domestic dietary needs or demands, this will require coordinated shifts, not only in land use but also in processing capacity, infrastructure, a decentralisation of market power away from a few large companies (Commerce Commission 2022) and market incentives. Grain‐milling capacity, for example, remains geographically concentrated—with major sites at three locations in Canterbury and smaller facilities in Wellington, Tirau, Mt Maunganui and Auckland. The lack of nearby processing infrastructure is a key barrier that discourages growers from producing milling‐quality wheat and pulses (McLeod et al. 2023). Investment in regional milling and storage and legume‐processing infrastructure would be necessary to unlock currently underutilised land and reduce reliance on imports. Without such infrastructural changes, farmers are unlikely to shift away from more reliable animal‐feed markets, even when land is biophysically capable of supplying human‐edible crops.

In the face of a food culture that is increasingly less healthy (Tomita et al. 2025), increased self‐sufficiency does not automatically translate into improved diet quality unless this is reflected in consumer demand (Millner and Roskruge 2013) and incomes that are adequate relative to food prices (Carter et al. 2010). Internationally, governments use targeted incentives, such as minimum prices, guaranteed public procurement (e.g., school lunches) and income supports, to stabilise production of nutrition‐critical crops (Table 8). Other options include commercial crop insurance (already widespread in North America and Australia) and targeted loans to improve access to capital and boost consumer demand in high‐value markets (Table 8).

**TABLE 8:** Examples of policy instruments used in various jurisdictions to incentivise the production of different foods for nutrition and food security.

Continent	Country/region	Staple/targeted crop(s)	Objectives	Policy/incentive mechanism	References
Africa	Botswana	Maize, cereals	Food security (minimum domestic production)	Government support programmes with climate‐adaptive policies	Masocha et al. (2024)
Africa	Ethiopia	Wheat	Food security (minimum domestic production)	Irrigation expansion, input subsidies, mechanisation support, guaranteed procurement	Demeke et al. (2014)
Africa	Ghana	Maize, cereals	Food security (minimum domestic production) and smallholder incomes	Subsidies for small/medium farms	Pauw (2022)
Africa	Malawi	Maize	Food security (minimum domestic production) and smallholder incomes	Subsidies (fertiliser + improved seed via vouchers) for smallholders	Chibwana et al. (2012)
Africa	Morocco	Cereals, legumes, vegetables	Food security (minimum domestic production) and smallholder incomes	Subsidised seeds, inputs, mechanisation, tariffs	Demeke et al. (2014)
Africa	Tanzania	Maize	Food security (minimum domestic production)	Subsidies for small/medium farms	Karata (2024)
Africa	Zambia	Maize, rice, millet, legumes	Food security (minimum domestic production) and smallholder incomes	Subsidised seeds, inputs, mechanisation and public procurement	Demeke et al. (2014)
Africa	Zimbabwe	Maize, wheat, soybeans	Food security (minimum domestic production) and smallholder incomes	Subsidised seeds, inputs, mechanisation and public procurement	Amankwah and Gwatidzo (2024)
Asia	China	Rice, wheat, maize	Food security (minimum domestic production)	Subsidies, insurance, minimum purchase prices	Wang et al. (2025)
Asia	India	Rice, wheat, pulses	Food security (minimum domestic production) and smallholder incomes	Minimum price, public procurement and distribution system	OECD (2025)
Asia	Indonesia	Rice, maize, sugar	Food security (minimum domestic production)	Minimum price, input subsidies and public procurement	OECD (2025)
Asia	Philippines	Rice	Food security (minimum domestic production)	Subsidised seeds, inputs and mechanisation support	Department of Agriculture (2025)
Asia	South Korea	Rice	Food security (minimum domestic production)	Price supports and area‐based incentives	OECD (2025)
Europe	European Union	Cereals, protein crops, oilseeds, legumes, pulses	Food security (minimum domestic production), stabilise incomes and aesthetics	Income support, payment for ecological performance, market intervention and protection	European Commission (2022, 2023)
North America	Canada	Wheat, canola, pulses	Food security (minimum domestic production), stabilise incomes	Income stabilisation, insurance and marketing support	OECD (2025)
North America	United States	Corn, wheat, soybeans, rice	Food security (minimum domestic production), stabilise incomes	Minimum price for commodities, crop insurance, marketing loans	Senate and House of Representatives in Congress of the United States of America (1965), OECD (2025)
South America	Brazil	Beans, rice, cassava	Food security (minimum domestic production), stabilise incomes	Government procurement	Demeke et al. (2014)
South America	Ecuador	Rice, beans, maize	Food security (minimum domestic production), stabilise incomes	Minimum price, input subsidies and public procurement	Ministry for Primary Industries (2025a)

In New Zealand, however, financial incentives alone may be insufficient without parallel advisory support for land‐use change. Current advisory systems are dominated by levy‐funded, sector‐specific bodies with few incentives to promote diversification, although this may change with the advent of a cross‐sector advisory support system from the Ministry for Primary Industries (Ministry for Primary Industries 2025a). Meaningful shifts would require integrated policies: investment in regional processing infrastructure; financial mechanisms to reduce growers’ risk; advisory services capable of supporting diversification; and public‐sector demand signals through procurement. Without these measures, it will be difficult to significantly increase the degree to which domestic food production aligns with domestic food consumption.

Perhaps more urgent is the need for sophisticated evaluation tools that allow us to assess the feasibility and consequences of actions intended to change food sourcing. Various tools have emerged to consider land use change in recent years (Saunders and Rutherford 2024), including some that link through to changes in national food consumption (McDowell et al. 2022). These have yet to examine localisation of food sourcing and the broader implications of ‘local' food systems, a discussion area that would benefit from quantitative simulation.

## 5 Conclusions

New Zealand possesses a substantial food production base and produces far more food than is required to meet domestic demand for most food groups. Projected increases in consumption, together with constrained production of cereals and legumes, nuts and seeds, indicate a growing reliance on imports for selected commodities; however, these deficits remain small relative to total national production and consumption. Furthermore, much of the projected increase in cereal demand appears to be linked to animal feed requirements rather than direct human consumption. Our analysis indicates that New Zealand has sufficient land resources to accommodate increased domestic self‐sufficiency, with relatively modest economic impacts associated with land‐use change. However, maintaining or expanding domestic production capacity is likely to depend less on land availability than on the alignment of processing infrastructure, storage, distribution networks, advisory support and market incentives with land‐use suitability. Assessing the implications of such changes will also require tools capable of linking the food system from production through processing, distribution and consumption, allowing evaluation of how land‐use change influences food resilience, dietary outcomes, supply security and broader sustainability objectives. The land‐suitability estimates should also be interpreted as dynamic, potentially changing in response to changes in temperature, drought, rainfall extremes and pest and disease pressures by 2045.

More broadly, improving food security in New Zealand is unlikely to be achieved solely through changes to domestic production. Food affordability and choice, equitable access, dietary quality and resilience to supply disruptions are all equally important components of food system security. Policies that support diversified production systems complemented by regional and local initiatives, regional processing capability and more competitive markets and which support households, families and whānau to make and follow through on informed choices about their diets may therefore provide greater long‐term benefits than focusing narrowly on self‐sufficiency.

## Funding

This work was supported by Ministry of Business, Innovation and Employment (MAUX2305).

## Conflicts of Interest

Note that R.W. McDowell is Editor‐in‐Chief of the Journal. Consequently, this submission was handled by an independent Editor.

## Supporting information


**Figure S1:** National map of land use suitability scores for apples. Values exceeding 0.6 and 0.8 were considered as suitable and high‐suitable, respectively. Data from Vetharaniam et al. (2022).
**Figure S2:** National map of land use suitability scores for avocados. Values exceeding 0.6 and 0.8 were considered as suitable and high‐suitable, respectively. Data from Vetharaniam et al. (2022).
**Figure S3:** National map of land use suitability scores for blueberries. Values exceeding 0.6 and 0.8 were considered as suitable and high‐suitable, respectively. Data from Vetharaniam et al. (2022).
**Figure S4:** National map of land use suitability scores for cherries. Values exceeding 0.6 and 0.8 were considered as suitable and high‐suitable, respectively. Data from Vetharaniam et al. (2022).
**Figure S5:** National map of land use suitability scores for chestnuts. Values exceeding 0.6 and 0.8 were considered as suitable and high‐suitable, respectively. Data from Thomas et al. (2021).
**Figure S6:** National map of land use suitability scores for citrus. Values exceeding 0.6 and 0.8 were considered as suitable and high‐suitable, respectively. Data from Thomas et al. (2021).
**Figure S7:** National map of land use dairy typologies as of 2023. Data from Monaghan et al. (2021).
**Figure S8:** National map of land use suitability scores for kiwifruit. Values exceeding 0.6 and 0.8 were considered as suitable and high‐suitable, respectively. Data from Vetharaniam et al. (2022).
**Figure S9:** National map of land use suitability scores for Macadamia nuts. Values exceeding 0.6 and 0.8 were considered as suitable and high‐suitable, respectively. Data from Thomas et al. (2021).
**Figure S10:** National map of land use suitability scores for maize. Values exceeding 0.6 and 0.8 were considered as suitable and high‐suitable, respectively. Data from Thomas et al. (2021).
**Figure S11:** National map of land use suitability scores for manuka honey. Values exceeding 0.6 and 0.8 were considered as suitable and high‐suitable, respectively. Data from Thomas et al. (2021).
**Figure S12:** National map of land use suitability scores for onions. Values exceeding 0.6 and 0.8 were considered as suitable and high‐suitable, respectively. Data from Thomas et al. (2021).
**Figure S13:** National map of land use suitability scores for peas. Values exceeding 0.6 and 0.8 were considered as suitable and high‐suitable, respectively. Data from Thomas et al. (2021).
**Figure S14:** National map of land use suitability scores for potatoes. Values exceeding 0.6 and 0.8 were considered as suitable and high‐suitable, respectively. Data from Thomas et al. (2021).
**Figure S15:** National map of land use suitability scores for wheat (left) barley (right). Values exceeding 0.6 and 0.8 were considered as suitable and high‐suitable, respectively. Data from Thomas et al. (2021).
**Figure S16:** National map of land use suitability scores for wine grapes (Sauvignon blanc). Values exceeding 0.6 and 0.8 were considered as suitable and high‐suitable, respectively. Data from Vetharaniam et al. (2022).

## Data Availability

The data that support the findings of this study are open and available from the corresponding author upon request.
