# Supplementary material for: The Cost of Self‐Sufficiency: Could New Zealand be Self‐Sufficient in Food Consumption?
Source: J R Soc N Z. 2026 Oct 10;56(5):e70083. doi: 10.1002/snz2.70083 (PMC13653447; doi:10.1002/snz2.70083)
Supplement: Supplementary file 1 [file SNZ2-56-e70083-s001.pdf]

Supplementary Information for:

The cost of self-sufficiency: could New Zealand be self-sufficient in food consumption?

R.W. McDowell[1,2,*], N.W. Smith[3,4], C. Eason[2,5], C. Smith[6]

[1] New Zealand Institute for Bioeconomy Sciences, Lincoln, New Zealand.

[2] Faculty of Agriculture and Life Sciences, Lincoln University, Lincoln, New Zealand.

[3] New Zealand Institute for Bioeconomy Sciences, Palmerston North, Christchurch, New Zealand.

[4] Sustainable Nutrition Initiative, Riddet Institute, Massey University, Palmerston North, New Zealand.

[5] AuOra Ltd, Wakatū Incorporation, Nelson, New Zealand.

[6] Kōtātā Insight Ltd.

* Corresponding author's email: richard.mcdowell@lincoln.ac.nz

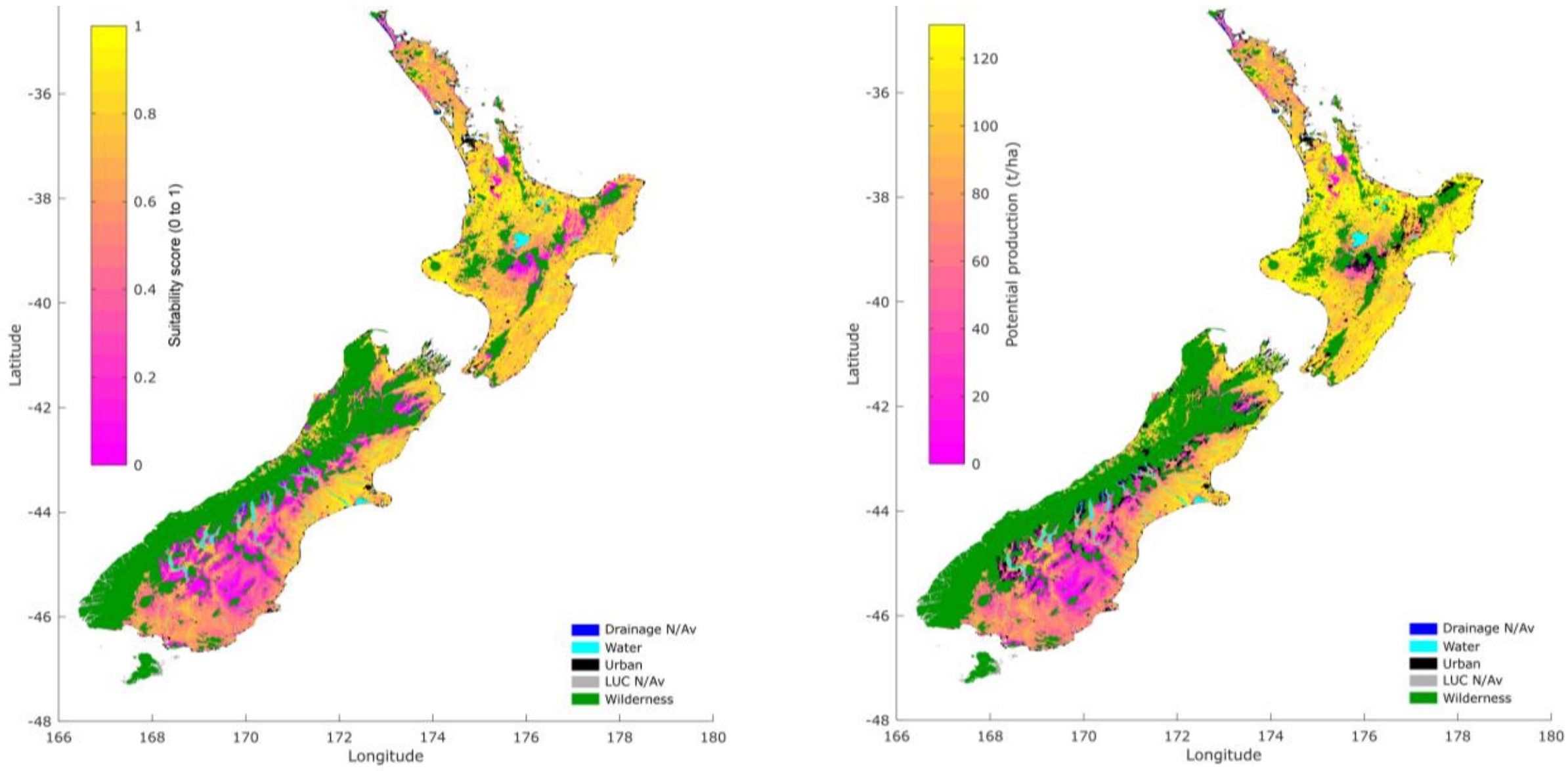

Supplementary Fig 1. National map of land use suitability scores for apples. Values exceeding 0.6 and 0.8 were considered as suitable and high-suitable, respectively. Data from Vetharaniam et al. (2022).

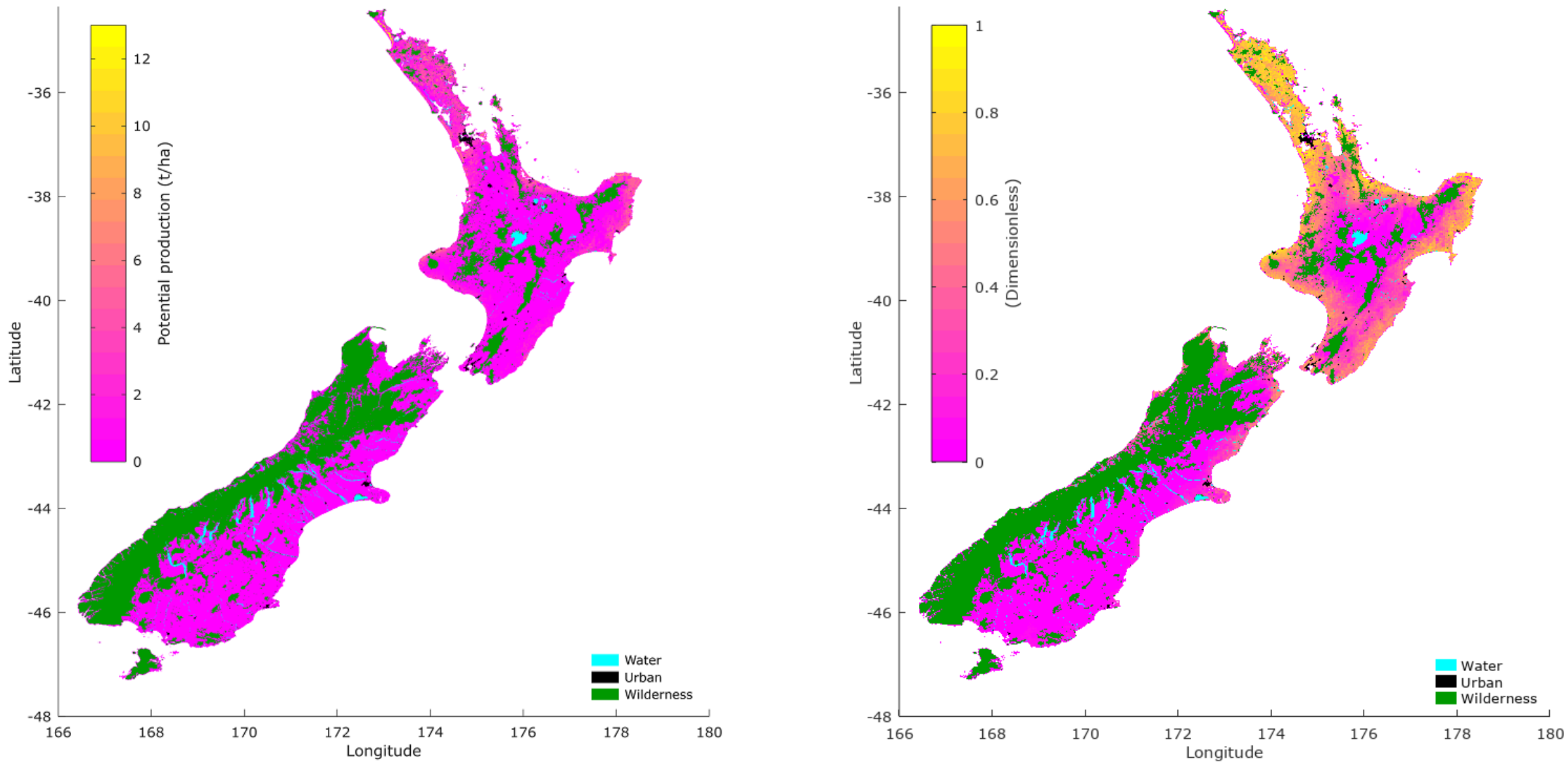

Supplementary Fig 2. National map of land use suitability scores for avocados. Values exceeding 0.6 and 0.8 were considered as suitable and high-suitable, respectively. Data from Vetharaniam et al. (2022).

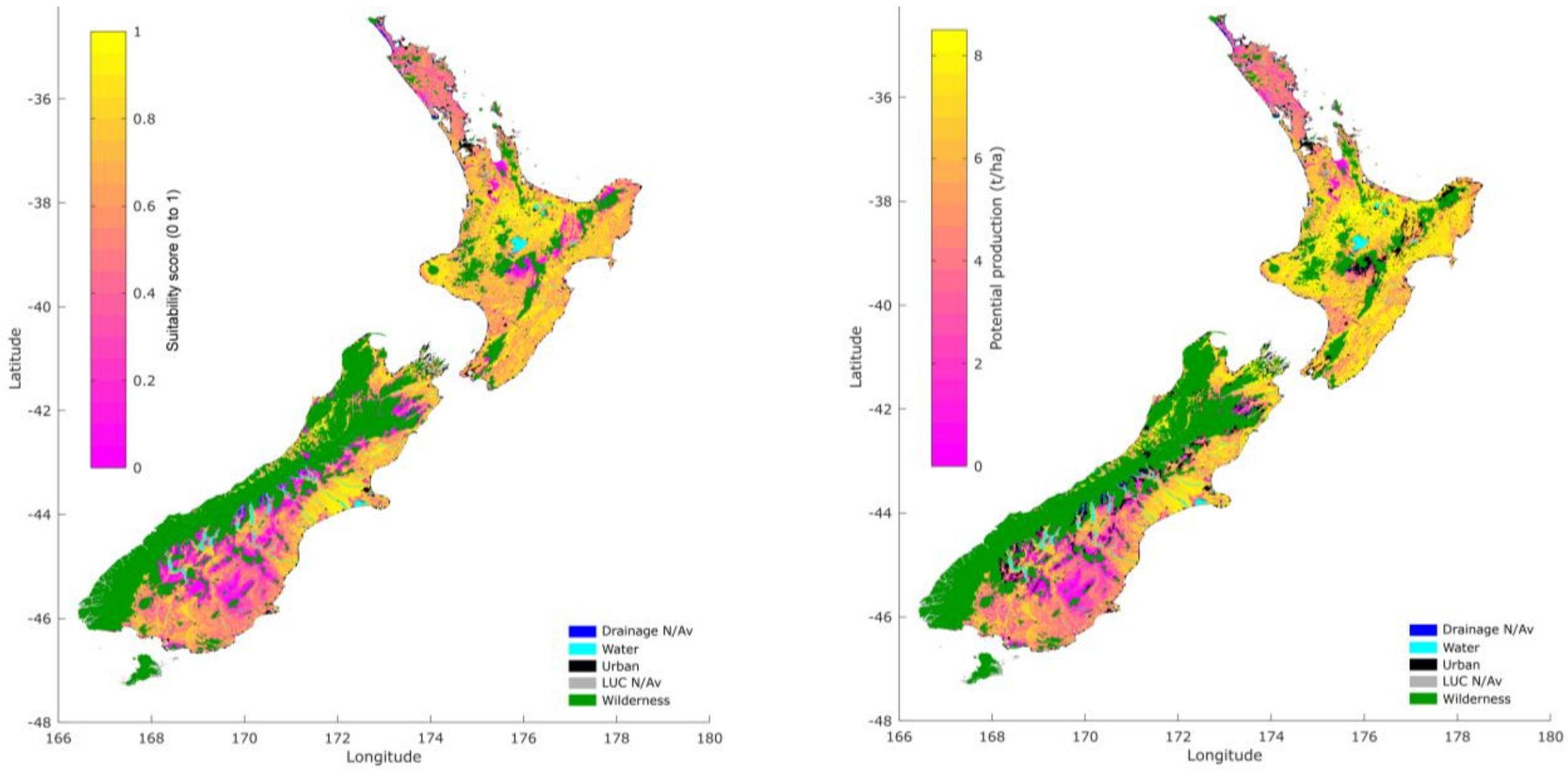

Supplementary Fig 3. National map of land use suitability scores for blueberries. Values exceeding 0.6 and 0.8 were considered as suitable and high-suitable, respectively. Data from Vetharaniam et al. (2022).

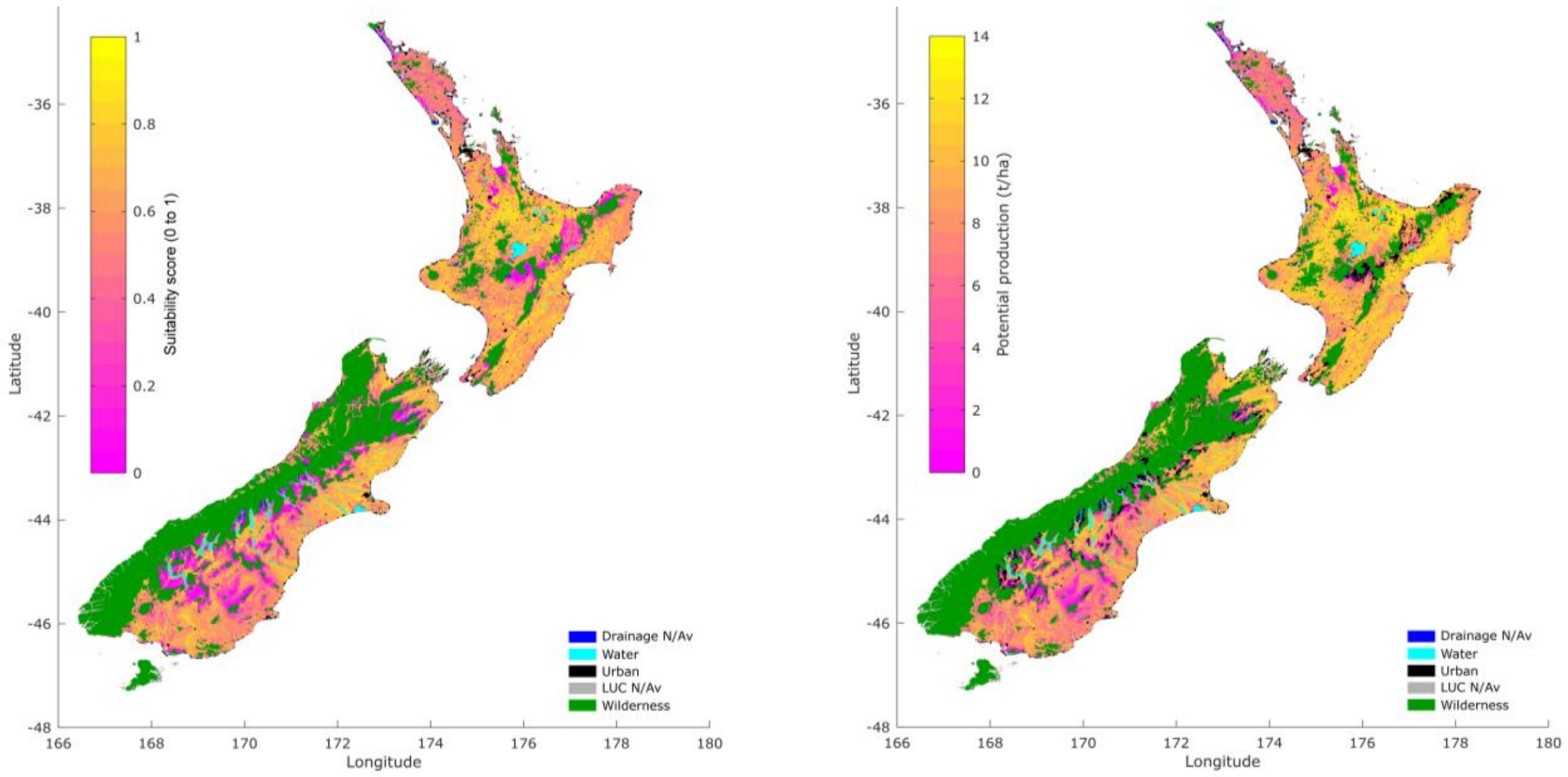

Supplementary Fig 4. National map of land use suitability scores for cherries. Values exceeding 0.6 and 0.8 were considered as suitable and high-suitable, respectively. Data from Vetharaniam et al. (2022).

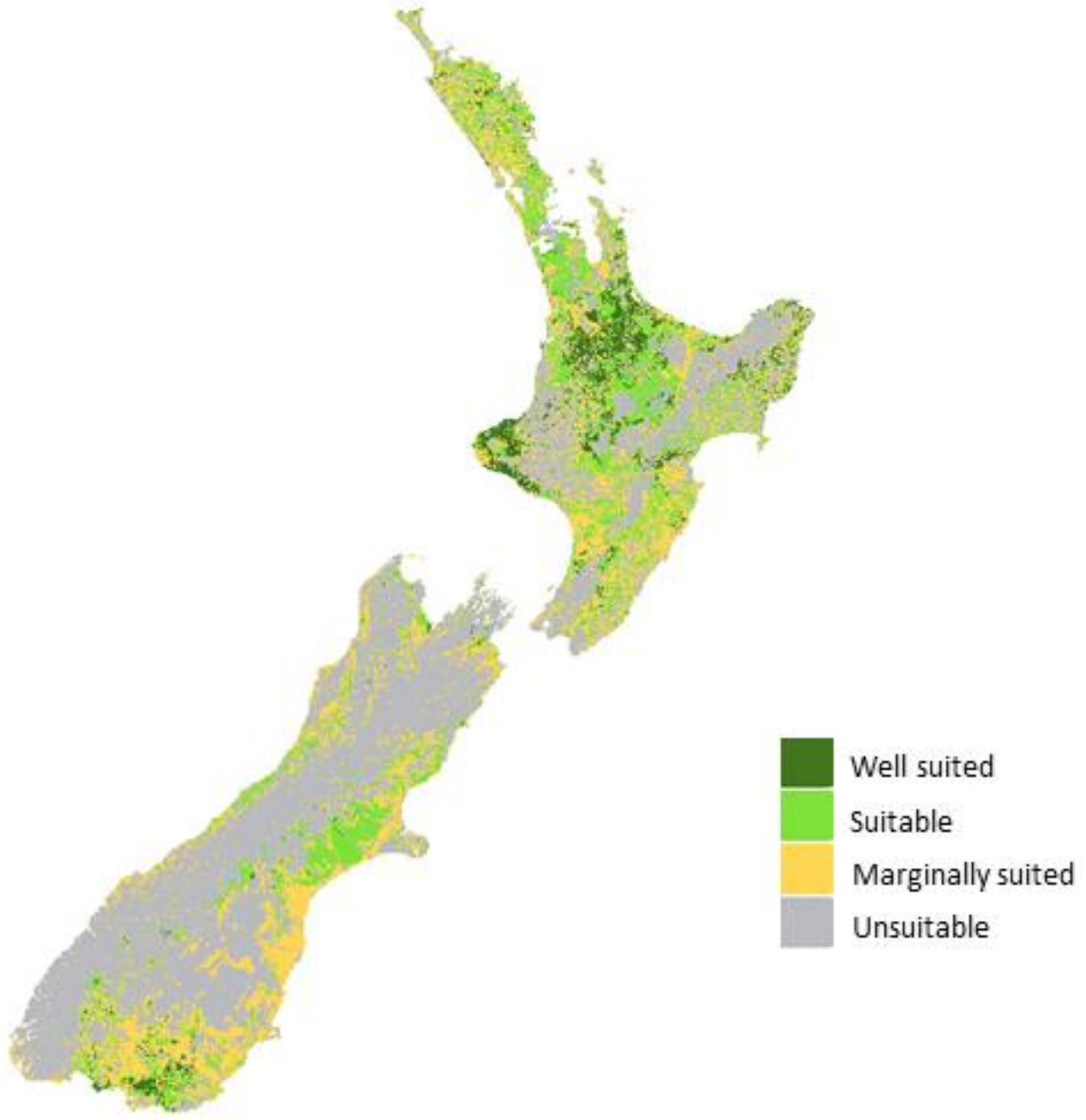

Supplementary Fig 5. National map of land use suitability scores for chestnuts. Values exceeding 0.6 and 0.8 were considered as suitable and high-suitable, respectively. Data from Thomas et al. (2021).

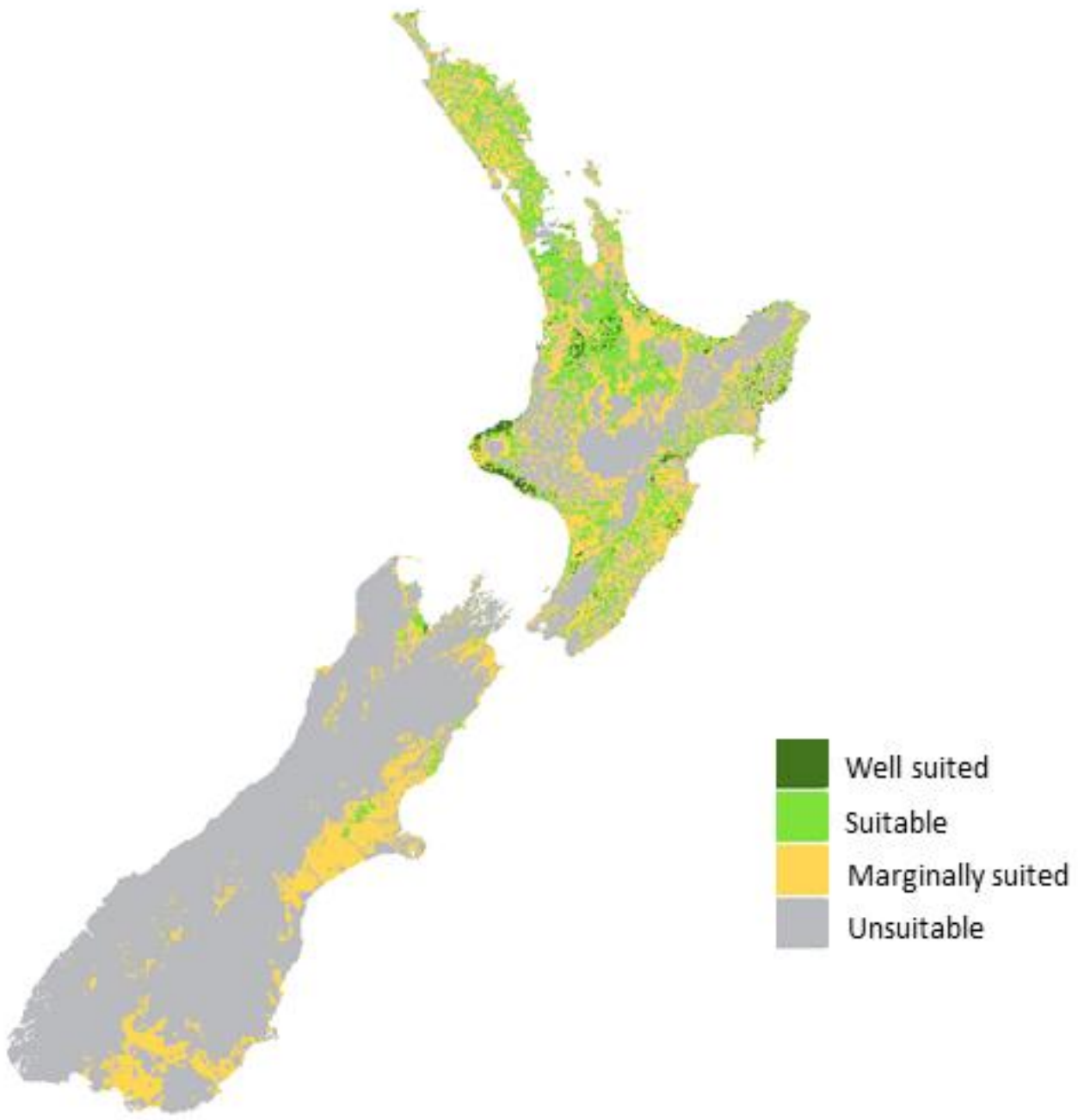

Supplementary Fig 6. National map of land use suitability scores for citrus. Values exceeding 0.6 and 0.8 were considered as suitable and high-suitable, respectively. Data from Thomas et al. (2021).

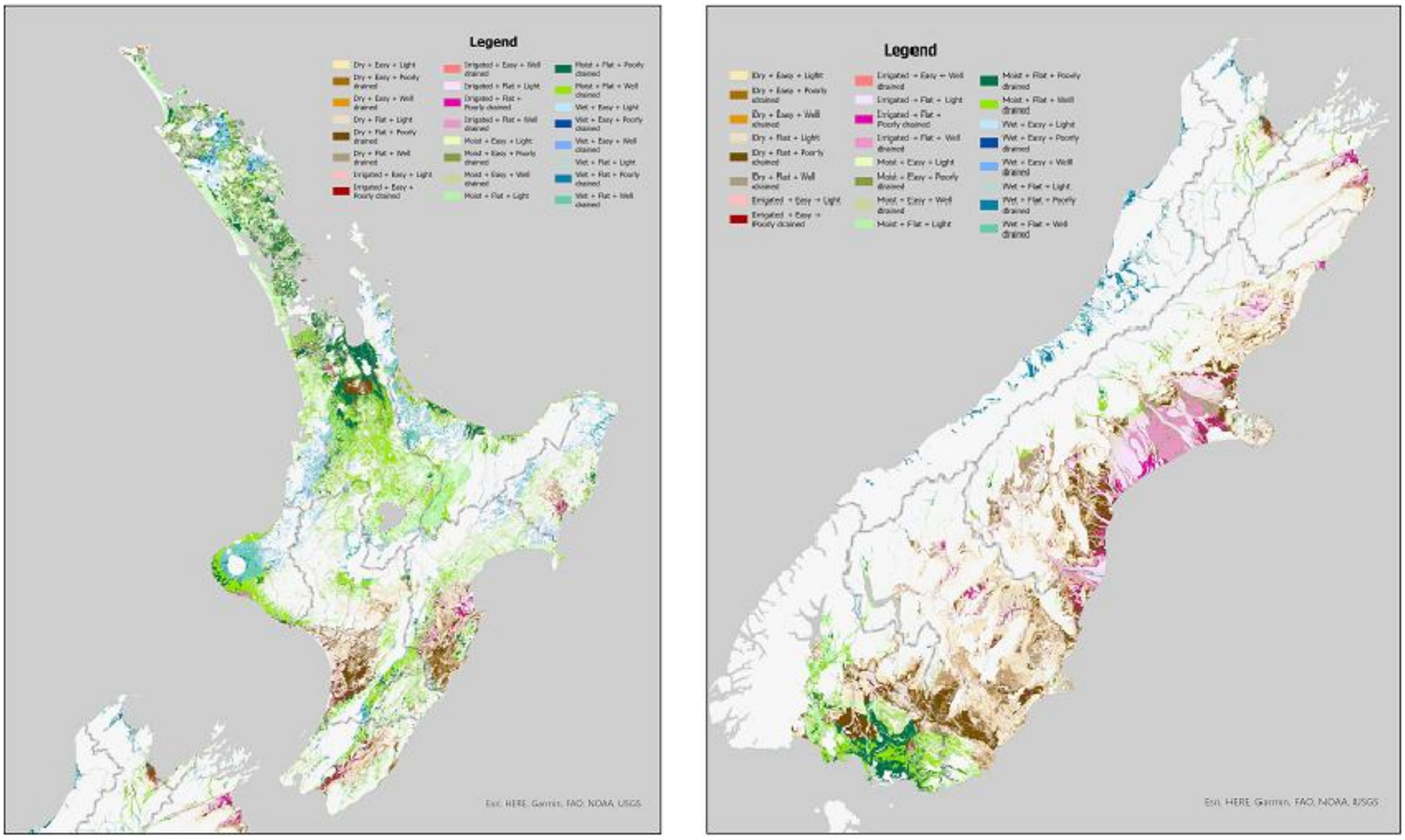

Supplementary Fig 7. National map of land use dairy typologies as of 2023. Data from Monaghan et al. (2021).

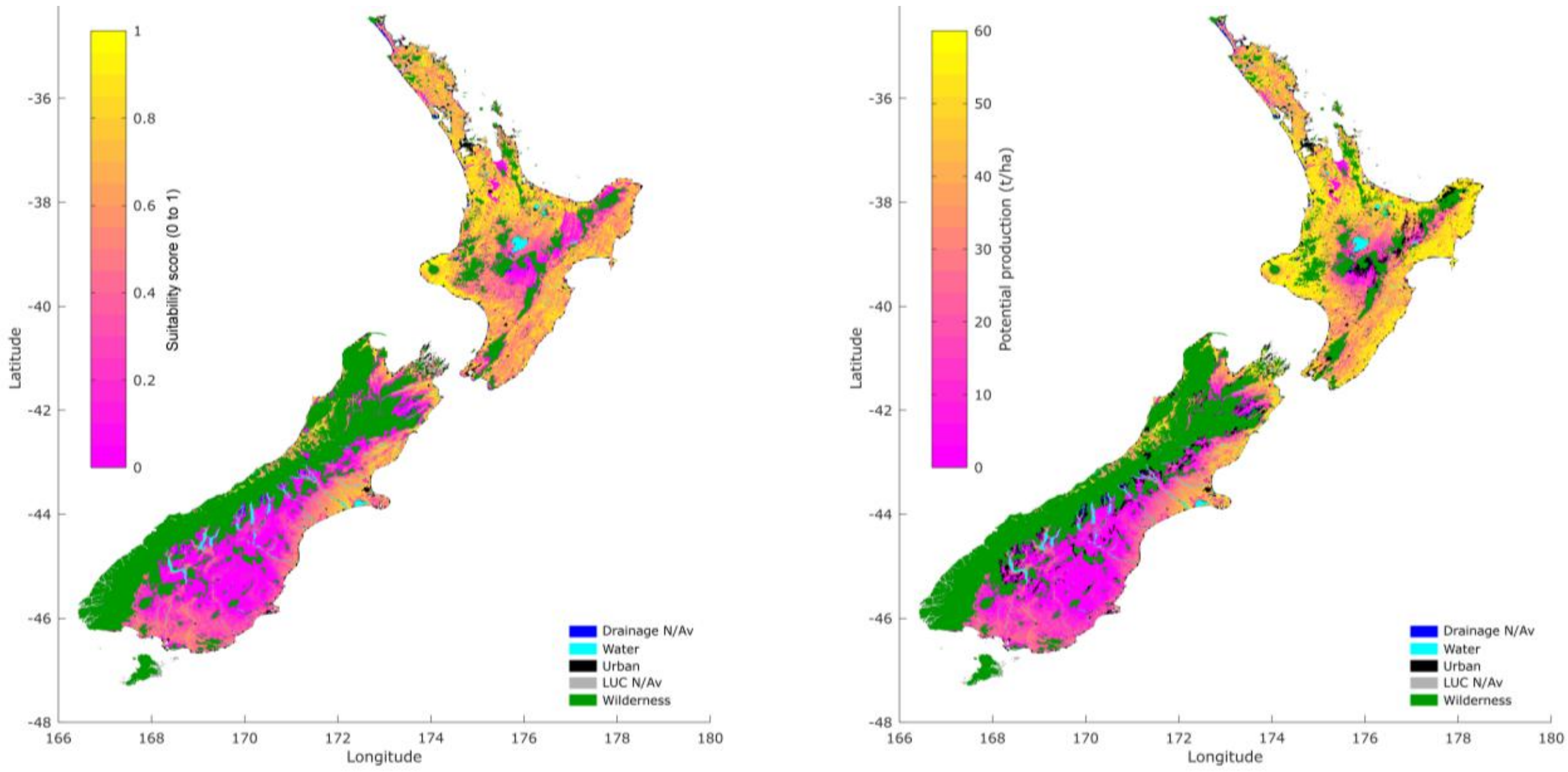

Supplementary Fig 8. National map of land use suitability scores for kiwifruit. Values exceeding 0.6 and 0.8 were considered as suitable and high-suitable, respectively. Data from Vetharaniam et al. (2022).

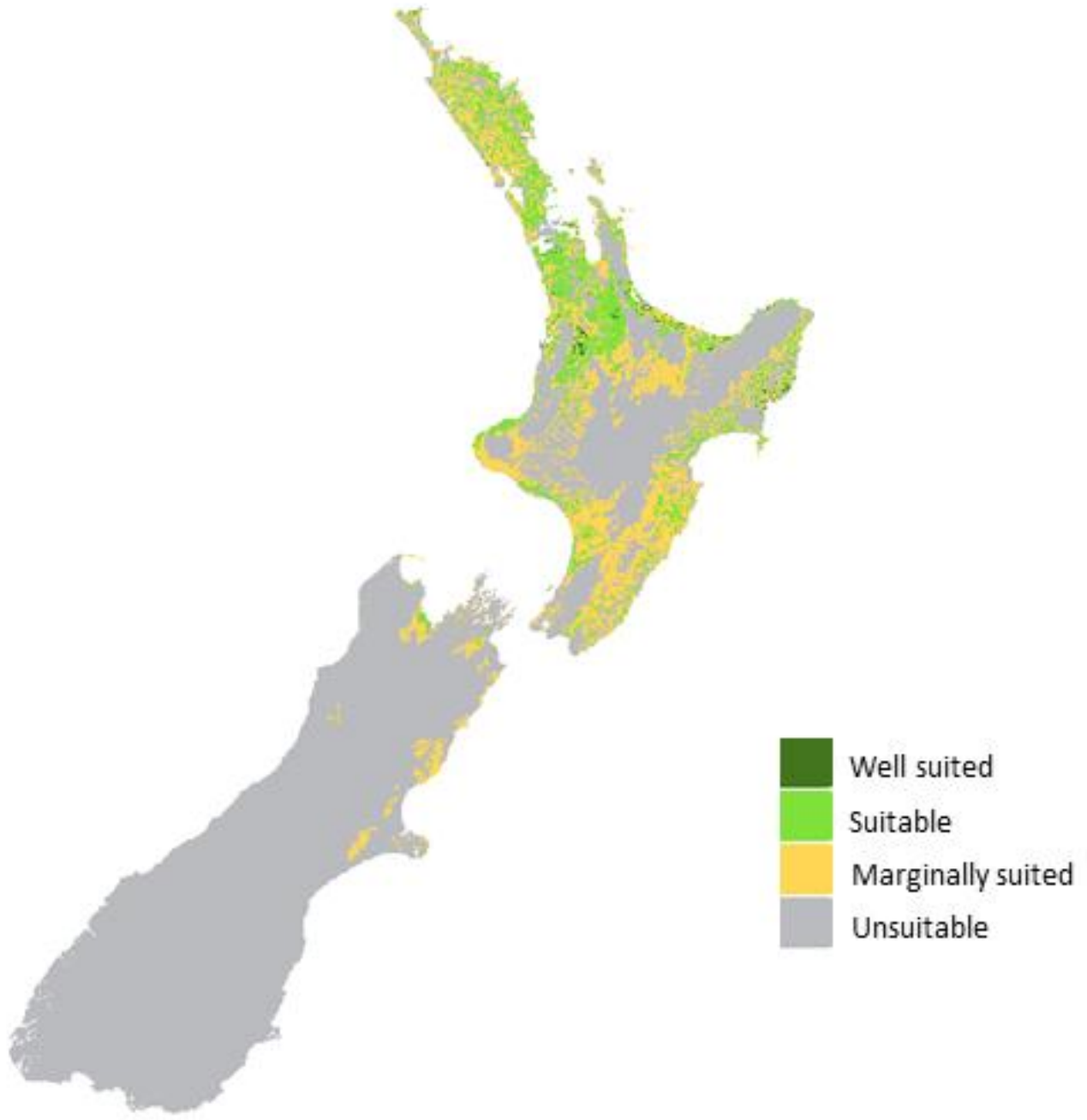

Supplementary Fig 9. National map of land use suitability scores for Macadamia nuts. Values exceeding 0.6 and 0.8 were considered as suitable and high-suitable, respectively. Data from Thomas et al. (2021).

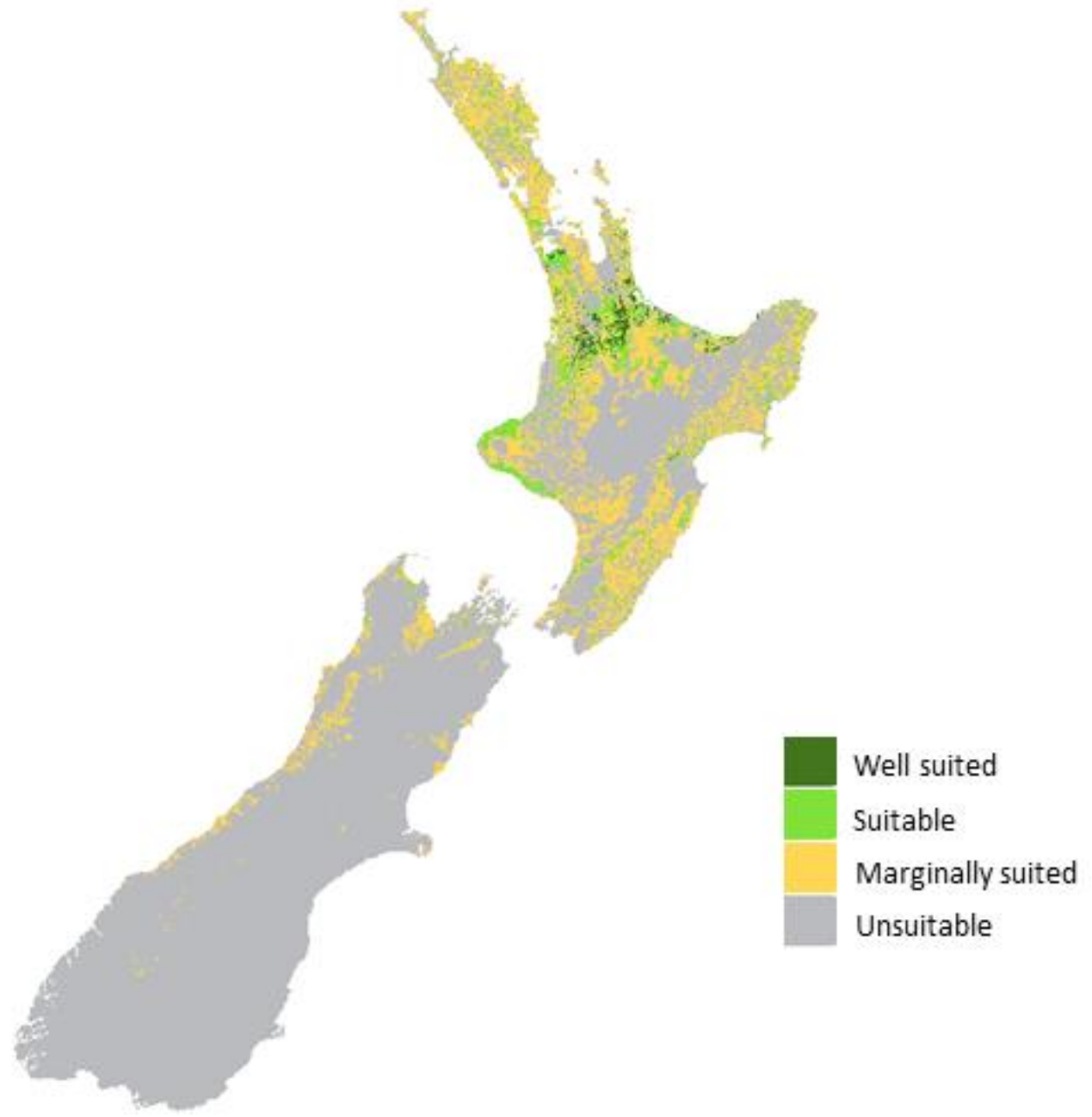

Supplementary Fig 10. National map of land use suitability scores for maize. Values exceeding 0.6 and 0.8 were considered as suitable and high-suitable, respectively. Data from Thomas et al. (2021).

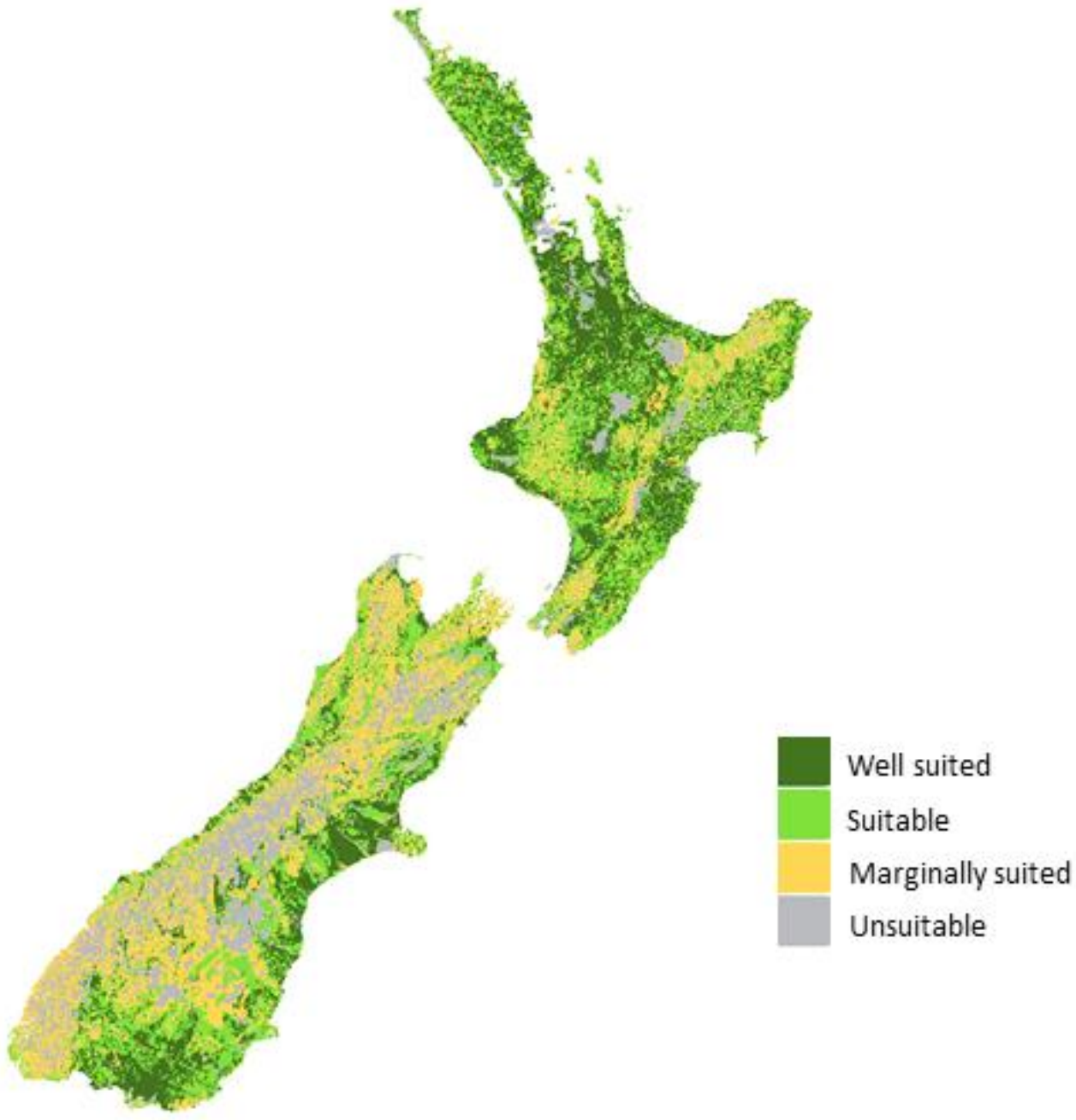

Supplementary Fig 11. National map of land use suitability scores for manuka honey. Values exceeding 0.6 and 0.8 were considered as suitable and high-suitable, respectively. Data from Thomas et al. (2021).

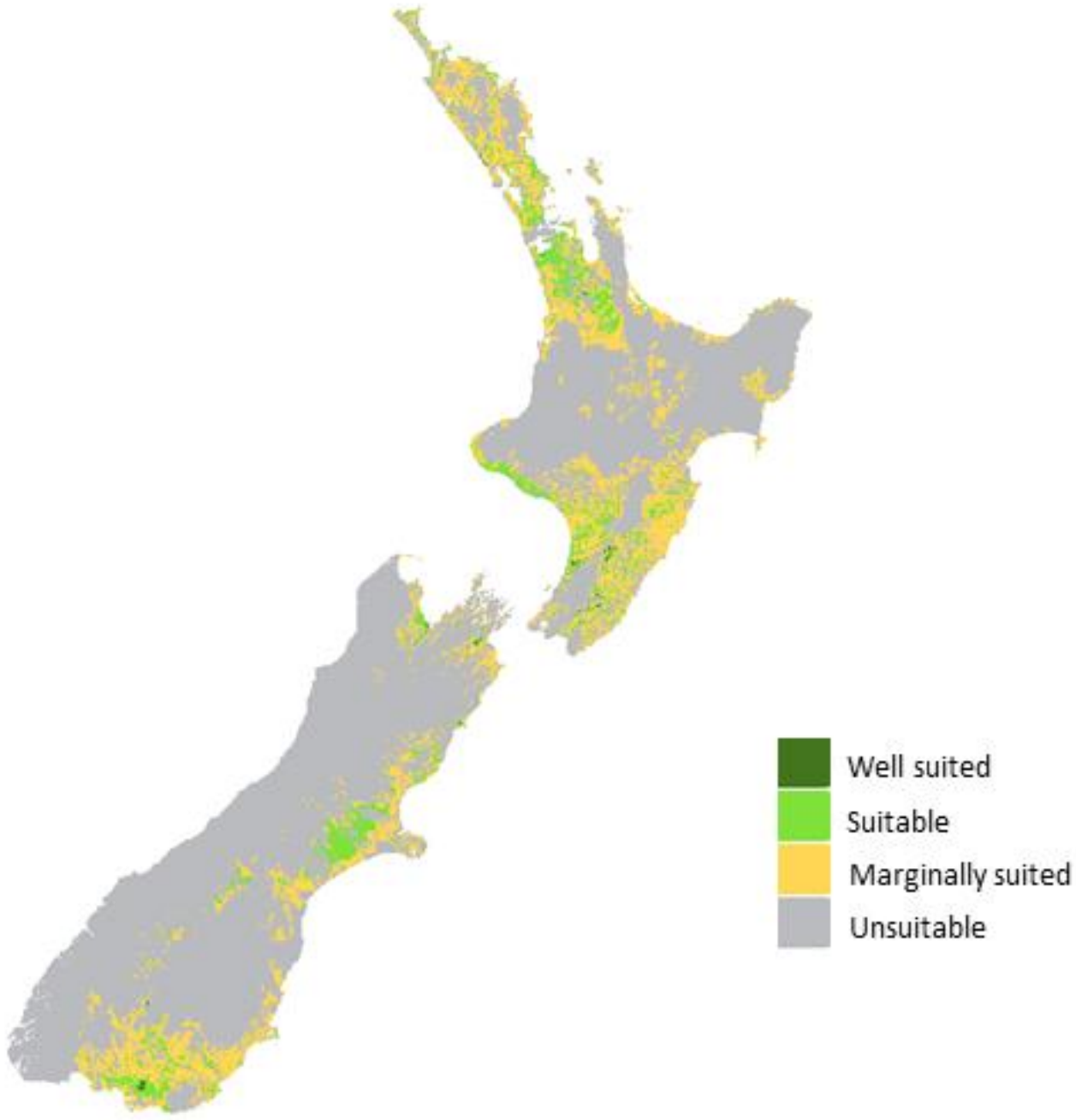

Supplementary Fig 12. National map of land use suitability scores for onions. Values exceeding 0.6 and 0.8 were considered as suitable and high-suitable, respectively. Data from Thomas et al. (2021).

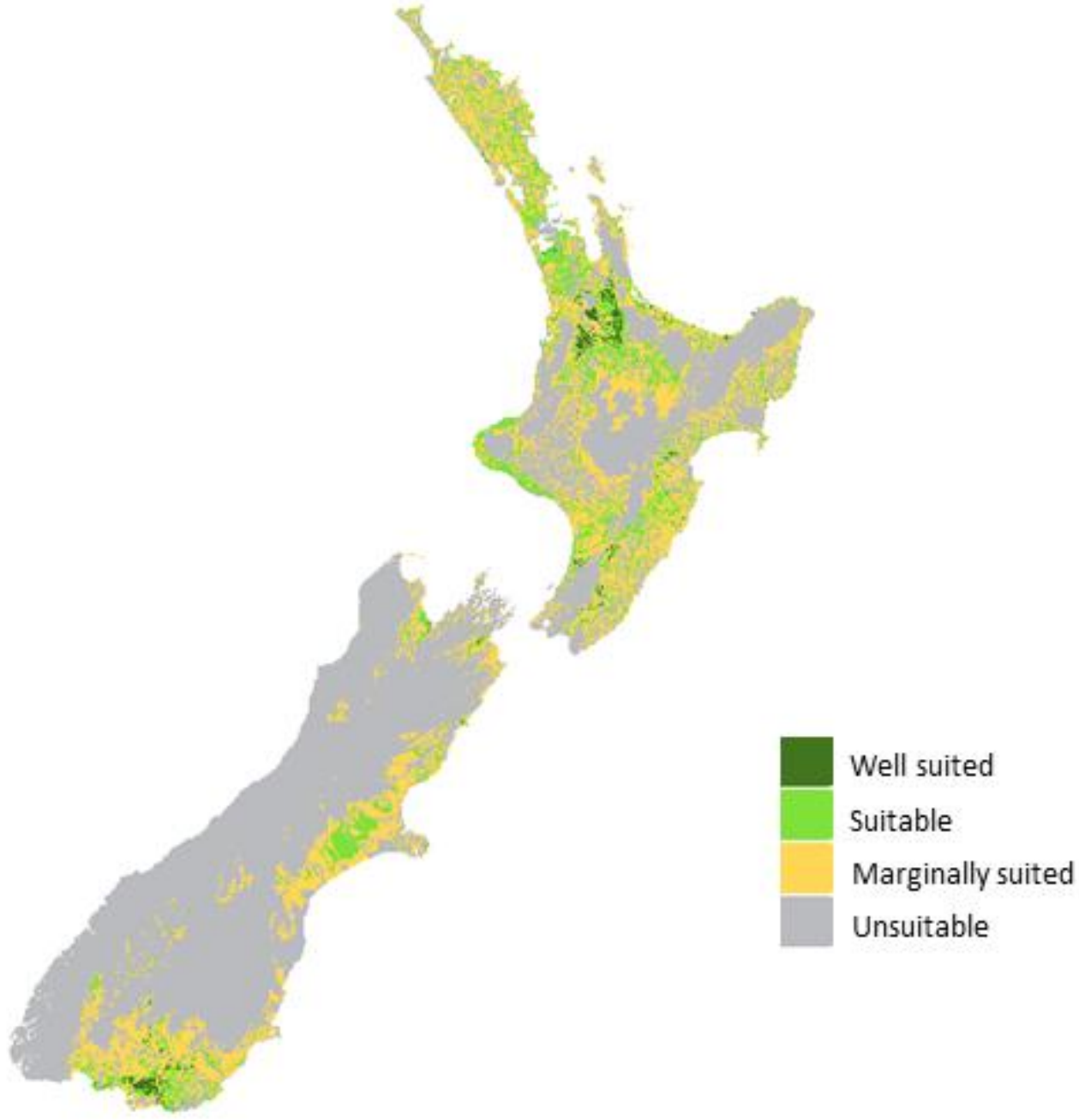

Supplementary Fig 13. National map of land use suitability scores for peas. Values exceeding 0.6 and 0.8 were considered as suitable and high-suitable, respectively. Data from Thomas et al. (2021).

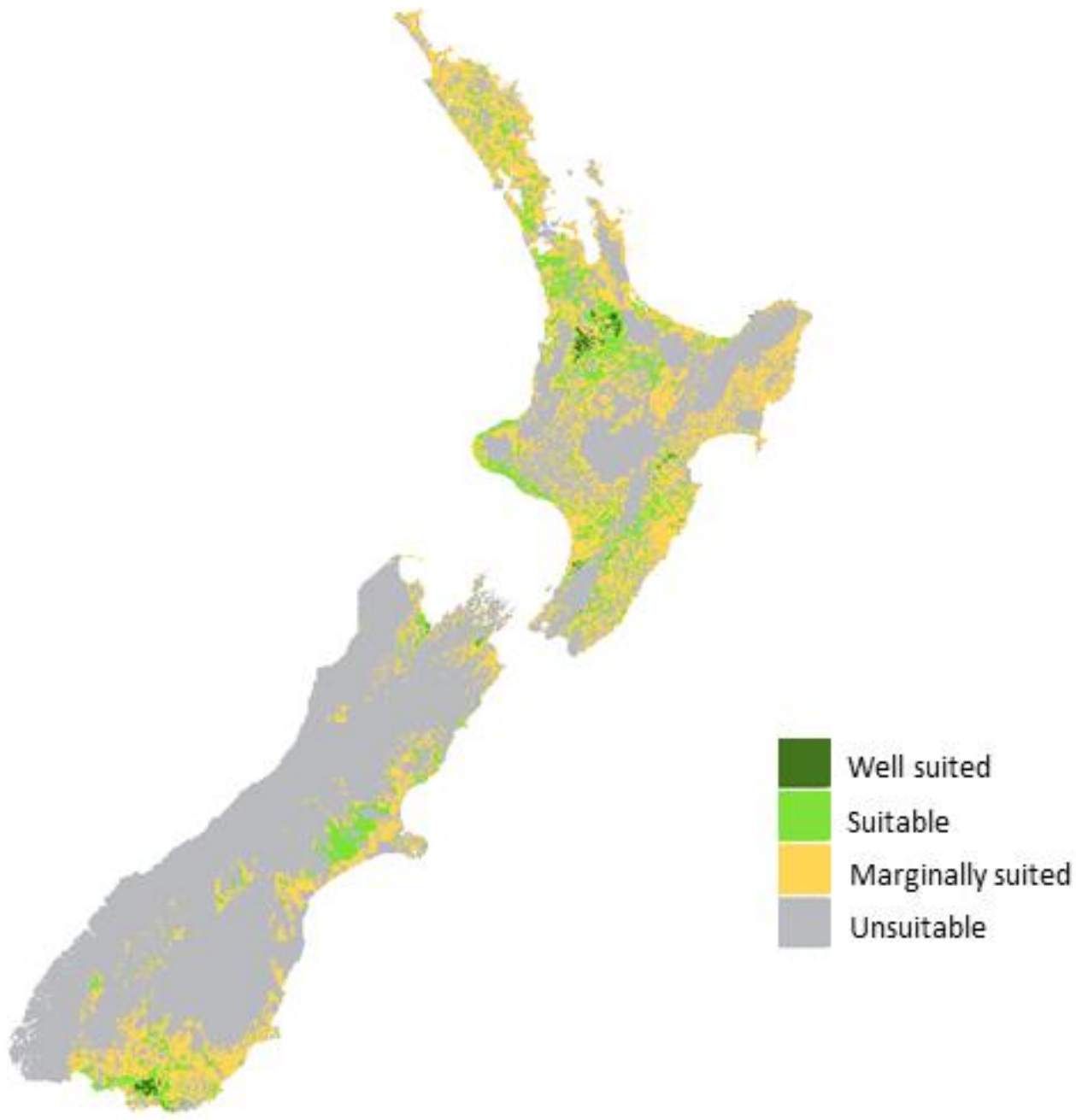

Supplementary Fig 14. National map of land use suitability scores for potatoes. Values exceeding 0.6 and 0.8 were considered as suitable and high-suitable, respectively. Data from Thomas et al. (2021).

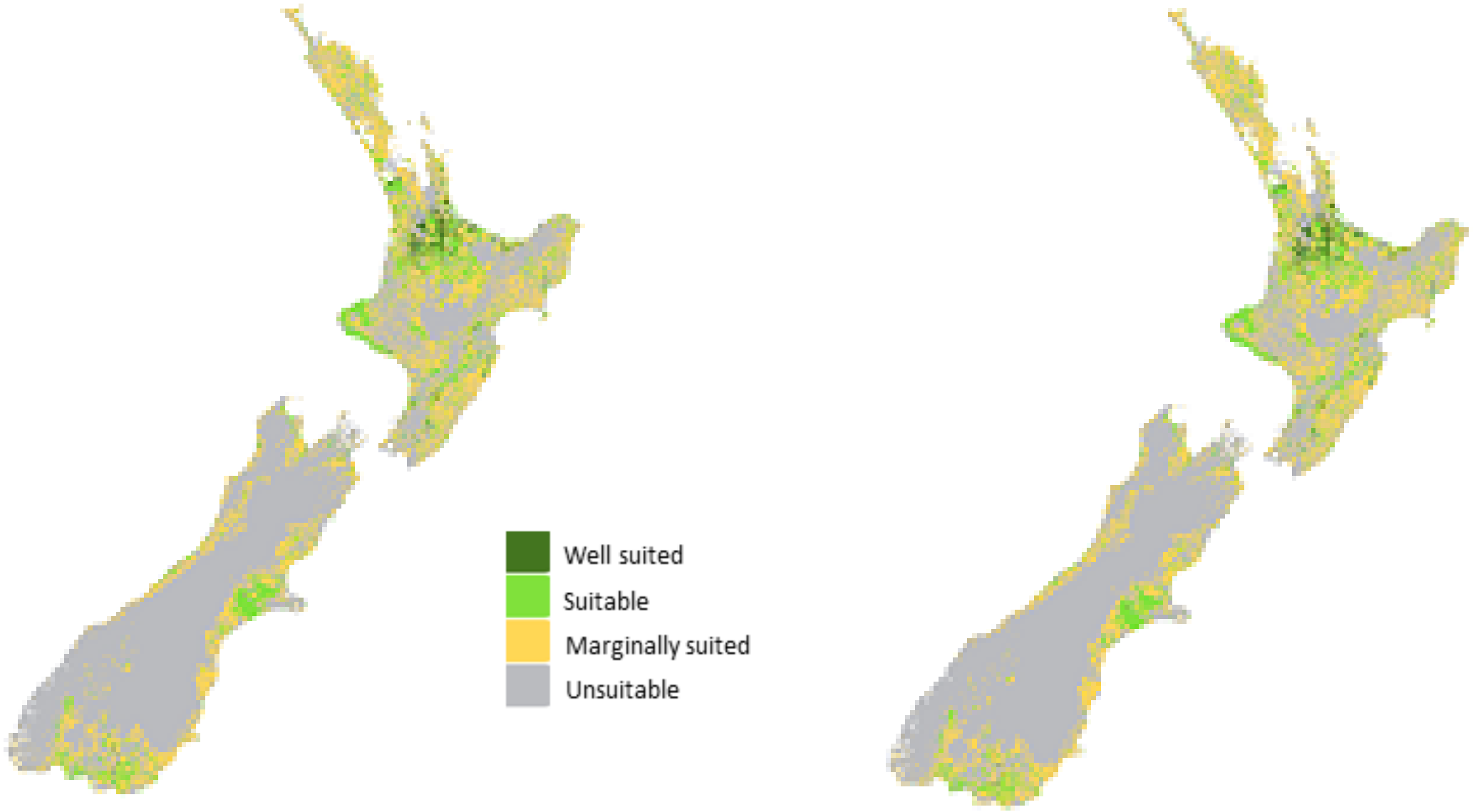

Supplementary Fig 15. National map of land use suitability scores for wheat (left) barley (right). Values exceeding 0.6 and 0.8 were considered as suitable and high-suitable, respectively. Data from Thomas et al. (2021).

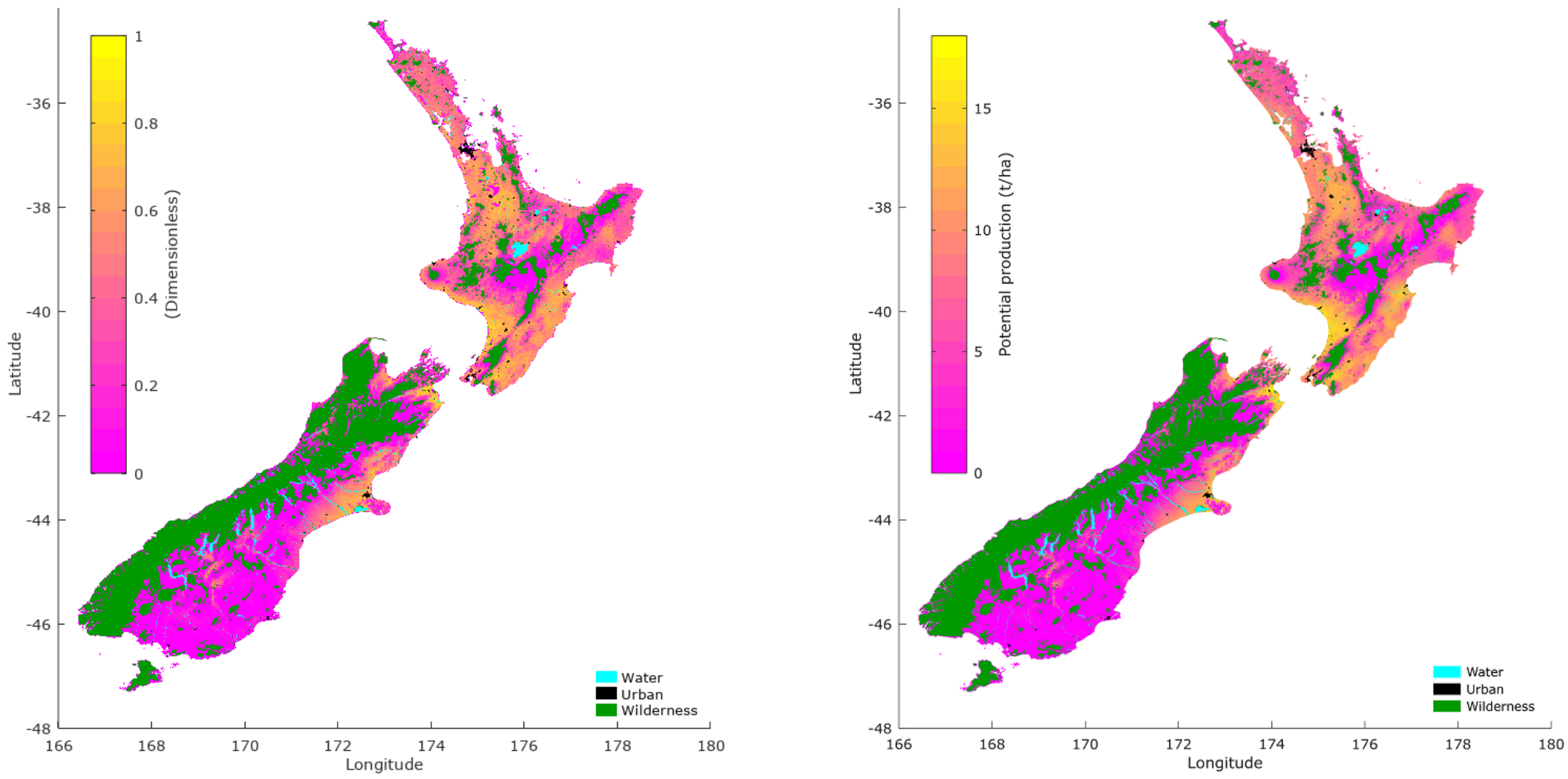

Supplementary Fig 16. National map of land use suitability scores for wine grapes (Sauvignon blanc). Values exceeding 0.6 and 0.8 were considered as suitable and high-suitable, respectively. Data from Vetharaniam et al. (2022).

References

Monaghan, R., Manderson, A., Basher, L., Smith, C., Burger, D., Meenken, E., McDowell, R., 2021. Quantifying contaminant losses to water from pastoral landuses in New Zealand I. Development of a spatial framework for assessing losses at a farm scale. N. Z. J. Agric. Res. 64, 344–364. https://doi.org/10.1080/00288233.2021.1936572

Thomas, S., Ausseil, A.-G., Guo, J., Herzig, A., Khaembah, E.N., Palmer, D., Renwick, A., Teixeira, E., van der Weerden, T., Wakelin, S.J., 2021. Evaluation of profitability and future potential for low

emission productive uses of land that is currently used for livestock (No. MPI Technical Paper No:2021/13). Ministry for Primary Industries, Wellington, New Zealand.
Vetharaniam, I., Müller, C., Stanley, J., Van Den Dijssel, C., Timar, L., Cummins, M., 2022. Modelling the effect of climate change on land suitability for growing perennial crops (No. MPI Technical Paper No: 2022/06). Ministry for Primary Industries, Wellington, New Zealand.
